# Two-Faced: Roles of JNK Signalling During Tumourigenesis in the *Drosophila* Model

**DOI:** 10.3389/fcell.2020.00042

**Published:** 2020-02-05

**Authors:** John E. La Marca, Helena E. Richardson

**Affiliations:** Richardson Laboratory, Department of Biochemistry and Genetics, La Trobe Institute for Molecular Science, La Trobe University, Melbourne, VIC, Australia

**Keywords:** JNK, *Drosophila*, tumourigenesis, *scrib*, Ras, apoptosis

## Abstract

The highly conserved c-Jun N-terminal Kinase (JNK) signalling pathway has many functions, regulating a diversity of processes: from cell movement during embryogenesis to the stress response of cells after environmental insults. Studies modelling cancer using the vinegar fly, *Drosophila melanogaster*, have identified both pro- and anti-tumourigenic roles for JNK signalling, depending on context. As a tumour suppressor, JNK signalling commonly is activated by conserved Tumour Necrosis Factor (TNF) signalling, which promotes the caspase-mediated death of tumourigenic cells. JNK pathway activation can also occur via actin cytoskeleton alterations, and after cellular damage inflicted by reactive oxygen species (ROS). Additionally, JNK signalling frequently acts in concert with Salvador-Warts-Hippo (SWH) signalling – either upstream of or parallel to this potent growth-suppressing pathway. As a tumour promoter, JNK signalling is co-opted by cells expressing activated Ras-MAPK signalling (among other pathways), and used to drive cell morphological changes, induce invasive behaviours, block differentiation, and enable persistent cell proliferation. Furthermore, JNK is capable of non-autonomous influences within tumour microenvironments by effecting the transcription of various cell growth- and proliferation-promoting molecules. In this review, we discuss these aspects of JNK signalling in *Drosophila* tumourigenesis models, and highlight recent publications that have expanded our knowledge of this important and versatile pathway.

## Introduction

**FIGURE 1:**
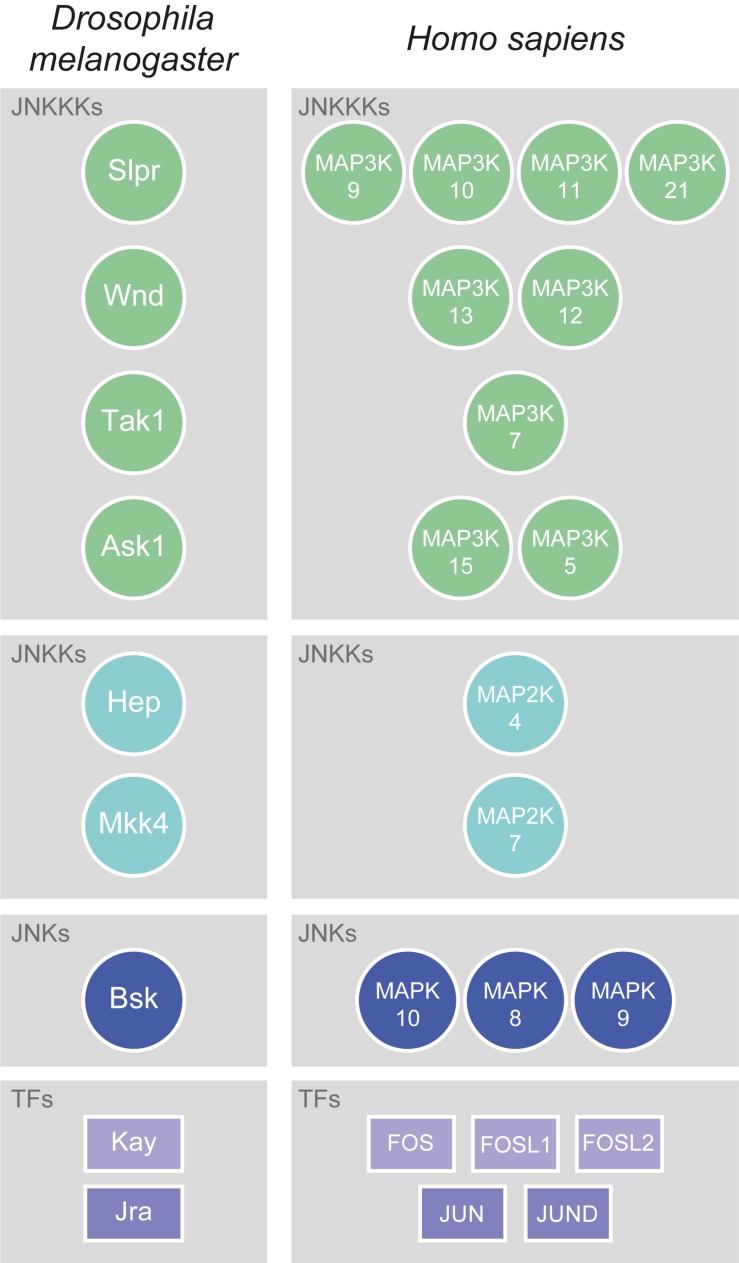
Conservation of JNK signalling core. The kinase core of JNK signalling is well-conserved between flies and mammals. In *Drosophila*, there are at least four JNKKKs: Slpr (Slipper), Wnd (Wallenda), Tak1 (TGFβ-associated kinase 1), and Ask1 (Apoptotic signal-regulating kinase 1). All have multiple human orthologues, which are Mitogen-activated protein kinase kinase kinases (MAP3Ks), with the most highly conserved being shown here. Slpr is most closely related to MAP3K9, MAP3K10, MAP3K11, and MAP3K21, Wnd to MAP3K13 and MAP3K12, Tak1 to MAP3K7, and Ask1 to MAP3K15 and MAP3K5. The *Drosophila* JNKKs are Hep (Hemipterous) and Mkk4 (MAP kinase kinase 4), which have human orthologues amongst the Mitogen-activated protein kinase kinases (MAP2Ks). Hep is most closely related to MAP2K4, and Mkk4 to MAP2K7. While *Drosophila* has only one JNK, Basket (Bsk), there are three conserved Mitogen-activated protein kinase (MAPK) orthologues to Bsk in humans: MAPK10, MAPK8, and MAPK9. JNKs upregulate the activity of various TFs, the best known of which are those that form the heterodimeric AP-1 complex. In *Drosophila*, those TFs are Kay (Kayak) and Jra (Jun-related antigen). In humans, the orthologues of Kay are FOS (Fos proto-oncogene, AP-1 transcription factor subunit), FOSL1 (FOS like 1, AP-1 transcription factor subunit), and FOSL2 (FOS like 2, AP-1 transcription factor subunit), while the orthologues of Jra are JUN (Jun proto-oncogene, AP-1 transcription factor subunit) and JUND (JunD proto-oncogene, AP-1 transcription factor subunit).

Jun N-terminal kinase signalling is a conserved Mitogen-Activated Protein Kinase (MAPK, N.B. for a glossary of abbreviated terms refer to Supplementary Table 1) signalling pathway which, through a conserved kinase cascade, acts to influence gene transcription, and hence the cellular response to various stimuli. In *Drosophila*, the sole JNK is Basket (Bsk; orthologue of human JNK1, JNK2, and JNK3, also known as MAPK8, MAPK9, and MAPK10, respectively), which acts to phosphorylate and activate a number of transcription factors (TFs). The best known JNK-activated TFs are Jun-related antigen (Jra) and Kayak (Kay; whose closest human orthologues are JUN (Jun proto-oncogene, AP-1 TF subunit) and FOS (Fos proto-oncogene, AP-1 TF subunit), respectively) (Figure 1), which together make up the heterodimeric Activator Protein-1 (AP-1) complex. While other Bsk targets also exist, upstream of Bsk the signalling network is much more complex. In order to activate JNK/Bsk there are at least two JNK kinases (JNKKs) – Hemipterous (Hep) and MAP kinase kinase 4 (Mkk4; orthologues of human MAP2K7 and MAP2K4, respectively) – and at least four JNKK kinases (JNKKKs) – Slipper (Slpr, human orthologues MAP3K9, MAP3K10, MAP3K11, and MAP3K21), Wallenda (Wnd, human orthologues MAP3K13 and MAP3K12), TGFβ-associated kinase 1 (Tak1, human orthologue MAP3K7), and Apoptotic signal-regulating kinase 1 (Ask1, human orthologues MAP3K15 and MAP3K5) (Figure 1). These kinases follow an ever-growing multitude of signalling pathways, molecules, and stimuli that feed into the activation of JNK. Despite this complexity, the kinase core of Hep-Bsk is generally considered to be the canonical and main effector of JNK signalling.

Originally identified in the Heidelberg genetic screens as a mutant that had improper dorsal closure during embryogenesis (Nüsslein-Volhard et al., 1984), it was not until a decade later that *bsk* was determined to be the orthologue of the mammalian JNK genes (Riesgo-Escovar et al., 1996; Sluss et al., 1996), a discovery that followed closely on the heels of the identification of *hep* as a JNKK (Glise et al., 1995). Since then, astonishingly large bodies of work have identified JNK signalling as being critical in a multitude of biological processes, such as regulating cell morphology and migration behaviours (via inducing the expression of genes like the actin cross-linker *cheerio* (*cher*) (Pastor-Pareja et al., 2004; Külshammer and Uhlirova, 2013), or by upregulating targets like the integrin-associated scaffolding protein Paxillin (Huang et al., 2003; Llense and Martín-Blanco, 2008; Leong et al., 2009)), regulating organ size (Willsey et al., 2016), and promoting cell death by upregulating genes like *head involution defective* (*hid*) and *reaper* (*rpr*) (Moreno et al., 2002; Luo et al., 2007).

With such diverse functionality, it is perhaps no surprise that JNK signalling has also emerged as a key player in tumourigenesis in *Drosophila*, something it shares with its mammalian orthologue (reviewed in Wagner and Nebreda, 2009; Wu et al., 2019). Almost all human orthologues of the core JNK signalling hierarchy have been implicated in multiple cancers, though their roles are often not well understood, and are often context dependent. We have summarised some of the recent literature concerning the links between JNK signalling and human cancer in our Supplementary Material (Supplementary Table 2). The role of JNK in tumourigenesis in flies is relatively better understood, but still exceedingly complex, with the pathway fulfilling different, seemingly opposing roles depending on the context. Simply put, JNK signalling is capable of both eliminating pre-tumourigenic cells via apoptosis, but also can cooperate with various genetic insults to promote tumourigenesis. In this review, we will examine these pro- and anti-tumourigenic roles of JNK signalling, non-autonomous roles of the pathway during tumourigenesis, and the various activation modes of the pathway in these contexts.

## Anti-Tumourigenic JNK Signalling

**FIGURE 2:**
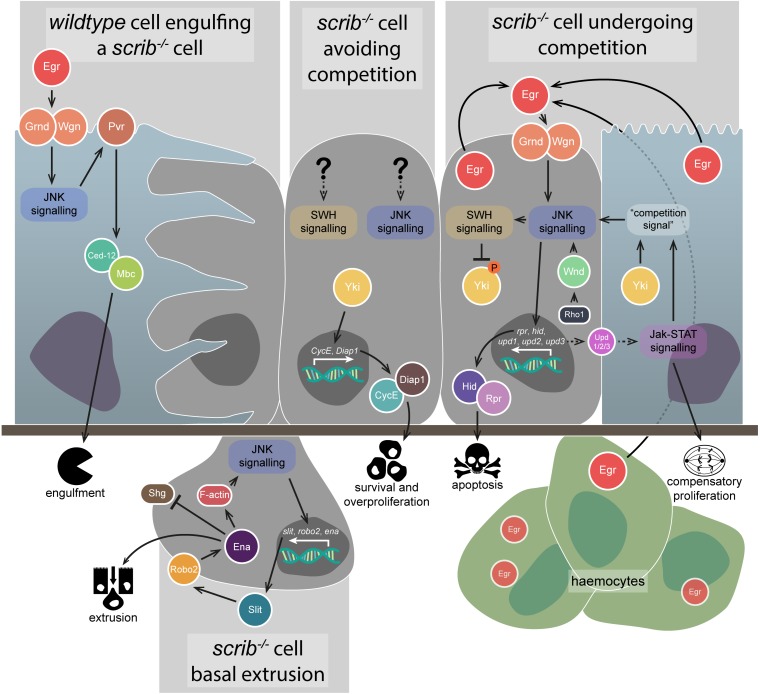
Anti-tumourigenic JNK signalling. JNK signalling has several different anti-tumourigenic roles, which are best understood in the context of *scrib*^–/–^ clone elimination from epithelial tissues. During cell competition, *scrib*^–/–^ cell elimination depends on JNK signalling (rightmost image) – the pathway is activated by both autocrine and paracrine Egr and promotes apoptosis via Hid and Rpr, as well as SWH signalling-mediated Yki downregulation. JNK signalling is also activated by Rho1 signalling through Wnd, and partly depends on an unknown “competition signal” from the *wildtype* neighbours, which itself depends on Yki and Jak-STAT signalling. Jak-STAT signalling is activated in *wildtype* neighbour cells by JNK-mediated Upd family ligand expression in the *scrib*^–/–^ cells, and contributes to their compensatory proliferation. Autocrine JNK signalling also promotes *scrib*^–/–^ cell extrusion from the epithelial layer (lower image) – upregulation of the Slit-Robo2-Ena pathway downregulates Shg (E-cadherin) and promotes detachment from the tissue, while also upregulating JNK via an F-actin-mediated feedback loop. The *wildtype* neighbour cells are also capable of actively eliminating the *scrib*^–/–^ cells (leftmost image) – JNK signalling activated by Egr promotes engulfment behaviours by the *wildtype* cells, activated via Pvr, Ced-12, and Mbc. However, if *scrib*^–/–^ cells can evade competition, JNK signalling and SWH signalling (if they occur) are not capable of downregulating Yki activity to a sufficient degree that the cells can be eliminated (central image) – instead, Yki promotes cell survival and overproliferation by upregulating targets such as CycE and Diap1. Gene and protein name abbreviations used in the diagram are as follows: Eiger (Egr), Grindelwald (Grnd), Wengen (Wgn), PDGF- and VEGF-receptor related (Pvr), Myoblast city (Mbc), Yorkie (Yki), *Cyclin E* (*CycE*), *Death-associated inhibitor of apoptosis 1* (*Diap1*), Wallenda (Wnd), *reaper* (*rpr*), *head involution defective* (*hid*), *unpaired 1* (*upd1*), *unpaired 2* (*upd2*), *unpaired 3* (*upd3*), *roundabout 2* (*robo2*), *enabled* (*ena*), Shotgun (Shg).

The anti-tumourigenic effect of JNK signalling ultimately induces cell death due to the upregulation of apoptosis-inducing genes like *hid* and *rpr*, and the activation of caspases (Moreno et al., 2002; Luo et al., 2007; Shlevkov and Morata, 2012; Li et al., 2019). One scenario where this role is well documented is upon the clonal disruption of cell polarity. Cell polarity is the asymmetric distribution of proteins within a cell, and the disruption of polarity is considered one of the hallmarks of cancer (Hanahan and Weinberg, 2011). In *Drosophila*, apico-basal polarity is critical to the proper formation of larval epithelial tissues, such as the wing and eye-antennal imaginal discs, and is controlled predominantly by the mutually antagonistic behaviours of three polarity protein modules: Scribble/Discs large 1/Lethal (2) giant larvae (Scrib/Dlg1/L(2)gl), Crumbs/Stardust/Patj (Crb/Sdt/Patj), and Bazooka/Par-6/atypical protein kinase C (Baz/Par-6/aPKC) (reviewed in Tepass, 2012). The largest body of work has examined Scrib/Dlg1/L(2)gl, where animals wholly mutant for any of these components produce neoplastic tumours, in which tissues overproliferate and show aberrant differentiation alongside a disorganised morphology – *scrib*, *dlg1*, and *l(2)gl* are therefore referred to as neoplastic tumour suppressor genes (nTSGs) (Bilder, 2004). However, while these wholly mutant tissues overgrow, clonal patches of epithelial tissue mutant for these genes are eliminated via a process termed cell competition. Cell competition is a surveillance mechanism that leads to the active elimination of cells that are “less fit” by their “more fit” neighbouring cells (reviewed in Fahey-Lozano et al., 2019; Ohsawa, 2019). Clones mutant for *scrib* (*scrib*^–/–^) are eliminated by apoptosis in *Drosophila* imaginal tissues, and this process is dependent on JNK signalling activity, as blocking JNK enables the cells to survive (Figure 2; Brumby and Richardson, 2003). These polarity mutant clones are therefore thought of as pre-tumourigenic, since if they are not removed tumours will develop. Furthermore, while *scrib*^–/–^ cells have enhanced proliferative capacity via JNK-independent upregulation of the cell cycle regulator, *Cyclin E* (*CycE*) (Brumby and Richardson, 2003; Leong et al., 2009), JNK signalling promotes their apoptosis, and the balance between these opposing phenotypes can be pushed in either direction by enhancing or disrupting JNK (Uhlirova and Bohmann, 2006).

Jun N-terminal kinase signalling was found to be primarily upregulated in cells at the borders of *scrib*^–/–^ clones and *wildtype* tissue, suggesting that its upregulation was not a direct consequence of *scrib* mutation (Leong et al., 2009). What, then, was the source? It was determined that JNK signalling, and the elimination of *scrib* or *dlg1* mutant clones, was dependent on activation of the pathway by TNF signalling – the *Drosophila* TNF, Eiger (Egr), binds to the TNF Receptors (TNFRs) Wengen (Wgn) and/or Grindelwald (Grnd), and eventually triggers activation of the kinase core of the JNK signalling pathway (Figure 2; Igaki et al., 2009; Andersen et al., 2015). Mislocalisation of Egr to endosomes within the *scrib*^–/–^ cells, rather than its upregulation, was determined to be the cause of the ectopic JNK signalling, with endocytosis increased in the clones – though, notably, endocytosis was only increased when *wildtype* tissue was adjacent to the *scrib*^–/–^ cells (Igaki et al., 2009). Although Egr was detectable in all the epithelial cells in the *scrib*^–/–^ mosaic tissue, genetic analyses showed that it acts in an autocrine manner within the *scrib*^–/–^ cells (Igaki et al., 2009), but this is unlikely to be the whole story – Egr was later shown to also be produced by haemocytes, circulating macrophage-like cells within the *Drosophila* haemolymph, and that its presence in these cells was sufficient for the activation of JNK in *scrib*^–/–^ cells (Figure 2; Vidal, 2010). While not investigated in *scrib*^–/–^ cells specifically, haemocyte attraction was shown to depend on JNK-mediated secretion of a cleaved form of the protein Tyrosyl-tRNA synthetase (Casas-Tintó et al., 2015). Additionally, *egr* is necessary for the elimination of *dlg1*-knockdown cells in wing imaginal discs, and for apoptosis within wholly *scrib* (or *dlg1*) mutant animals (Cordero et al., 2010). Regardless of the source of Egr, JNK signalling has a key role in eliminating *scrib*^–/–^ cells during cell competition by promoting apoptosis – however, blocking apoptosis in these *scrib*^–/–^ clones is not, in fact, as effective at preventing their elimination (and thus promoting tumourigenesis) as simply blocking JNK signalling, suggesting other removal mechanisms are at play (Brumby and Richardson, 2003). To wit, JNK signalling has been demonstrated to upregulate the genes *slit*, *roundabout 2* (*robo2*), and *enabled* (*ena*), which together act to promote *scrib*^–/–^ cell extrusion from the tissue – Sli-Robo2-Ena signalling disrupts Shotgun (shg, a.k.a. E-cadherin), and also forms a positive feedback loop with JNK signalling by promoting F-actin accumulation (Figure 2; Vaughen and Igaki, 2016).

Jun N-terminal kinase signalling is not exclusively active within polarity-impaired cells during cell competition. The pathway has also been shown to be active within their *wildtype* neighbours – Egr-dependent JNK activation in the *wildtype* cells promotes signalling via PDGF- and VEGF-receptor related (Pvr), which in turn activates Ced-12 and Myoblast city (Mbc) to promote engulfment and removal of the mutant cells by their healthy neighbours (Figure 2; Ohsawa et al., 2011). Furthermore, mechanisms have been identified that are involved in the recognition of polarity-impaired cells. Protein tyrosine phosphatase 10D (Ptp10D) is expressed on the surface of *scrib*^–/–^ cells, and is bound and activated by the ligand Stranded at second (Sas) expressed on the surface of their *wildtype* neighbours (Yamamoto et al., 2017). Activated Ptp10D suppresses epidermal growth factor receptor (Egfr) activity, allowing JNK signalling to act in its anti-tumourigenic capacity (Yamamoto et al., 2017). If Egfr activity were permitted due to *sas* or *Ptp10D* downregulation, activated Ras-MAPK signalling would occur alongside JNK signalling, the consequences of which we will discuss in a later section (“Pro-tumourigenic JNK signalling”).

Interestingly, *l(2)gl* mutant (*l(2)gl^–/–^*) clones and tissues behave somewhat differently to *scrib*^–/–^ cells, though they also upregulate JNK signalling, and are eliminated by JNK-dependent apoptosis (Froldi et al., 2010; Grzeschik et al., 2010; Menéndez et al., 2010; Tamori et al., 2010). Autocrine Egr is dispensable in *l(2)gl^–/–^* clones, as they still upregulate JNK signalling even when *egr* is knocked down in these cells; however, it is thought that *l(2)gl^–/–^* tissue growth and survival is more dependent on levels of the oncogenic TF Myc than on JNK signalling (Froldi et al., 2010).

As mentioned, *scrib*^–/–^ clones exhibit ectopic proliferation, but their potential to overgrow is modulated by JNK signalling-induced apoptosis. Inhibiting JNK allows these clones to overgrow, but where does this capability come from? One important growth regulating pathway is the SWH signalling pathway, which is a conserved inhibitor of tissue growth, which functions by phosphorylating and thus cytoplasmically sequestering the TF coactivator, Yorkie (Yki). This prevents Yki from interacting with and activating the TF Scalloped (Sd, a TEAD family TF) and its target genes, which include the cell cycle regulator *CycE* and the apoptosis inhibitor *Death-associated inhibitor of apoptosis 1* (*Diap1*) (reviewed in Misra and Irvine, 2018). Many reports have indicated that both the JNK and SWH signalling pathways are interwoven but elucidating exactly how they interact in polarity-impaired tumours has been difficult. Initial experiments in eye-antennal imaginal disc *scrib*^–/–^ clones indicated that reporters for certain Yki target genes were highly expressed (though variable), suggesting some level of SWH inhibition may be in effect and, indeed, *scrib*^–/–^ cell overproliferation was found to depend on Yki and Sd activity – however, blocking JNK signalling resulted in SWH inhibition, as revealed by the upregulation of Yki targets (Doggett et al., 2011). More light was shed on these results in a later study, which more closely examined SWH signalling in *scrib*^–/–^ clones in both eye-antennal and wing imaginal discs and found that Yki activity was specifically dependent on whether cell competition was occurring (Chen et al., 2012). Specifically, *scrib*^–/–^ clones not facing cell competition (by artificially lowering the fitness of their neighbours, or in wholly *scrib*^–/–^ tissue) showed elevated Yki activity and the cells overgrew, but in *scrib*^–/–^ cells undergoing competition Yki activity was downregulated, and this downregulation was mediated by JNK signalling (though other mechanisms are likely to contribute) (Figure 2; Chen et al., 2012). Notably, Yki activation is also required in the *wildtype* neighbours for their compensatory proliferation, where it is thought to act parallel to Janus kinase-Signal Transduction and Activator of Transcription (Jak-STAT) signalling to promote the elimination of the *scrib*^–/–^ cells (Figure 2; Chen et al., 2012; Schroeder et al., 2013).

Somewhat incongruous with these results is data from wing imaginal disc regions (not clones), where the induction of apoptosis in regions of disc tissue induced Yki activation in adjacent cells to promote compensatory proliferation (Sun and Irvine, 2011). Here, Yki activation was dependent on JNK signalling and, indeed, JNK signalling initiation was sufficient to induce Yki activity (Sun and Irvine, 2011). Interestingly, initiating neoplastic growth via the knockdown of *dlg1*/*l(2)gl* in large tissue regions also upregulated Yki activity and, in these instances, Yki upregulation was again dependent on JNK activity – possibly this is similar to the aforementioned *scrib*^–/–^ clones when dodging cell competition (Sun and Irvine, 2011). JNK signalling in these contexts may mediate Yki activation via downregulation of SWH signalling at the level of the Warts (Wts) protein kinase – JNK activity phosphorylates Ajuba LIM protein (Jub), which in turn binds and inactivates Wts (Sun and Irvine, 2013).

Interactions between JNK signalling and SWH/Yki in the context of JNK acting in an anti-tumourigenic role are not limited to the context of disrupted cell polarity. Inducing cytokinesis failure in wing imaginal disc cells promotes aneuploidy, which can lead to tumourigenesis, but JNK signalling is also upregulated in these cells and acts to downregulate Diap1 and a cell cycle regulator, String (Stg, orthologue of the human CDC25 proteins), thus promoting cell death and suppressing cell proliferation (Gerlach et al., 2018). However, SWH inhibition or Yki activation can bypass this JNK-mediated tumourigenesis prevention by facilitating Diap1 and Stg upregulation (Gerlach et al., 2018).

Overall, JNK signalling, primarily in its capacity as a pro-apoptotic regulator, plays a fundamental role as an anti-tumourigenic signal. This is particularly apparent (and best studied) in the context of polarity-deficient pre-tumourigenic cells, where it acts both autonomously and non-autonomously in facilitating their elimination. Furthermore, JNK signalling has a complex relationship with the SWH signalling pathway, where its interactions vary depending on context – in polarity-impaired, pre-tumourigenic cells, activated JNK signalling suppresses Yki activity (Doggett et al., 2011; Chen et al., 2012), whereas in regenerating wing imaginal disc tissue JNK signalling suppresses SWH signalling, and hence promotes Yki activity (Sun and Irvine, 2011). This role in promoting Yki activity is a hint at the two-faced nature of JNK signalling – depending on context, it can also be pro-tumourigenic.

## Pro-Tumourigenic JNK Signalling

**FIGURE 3:**
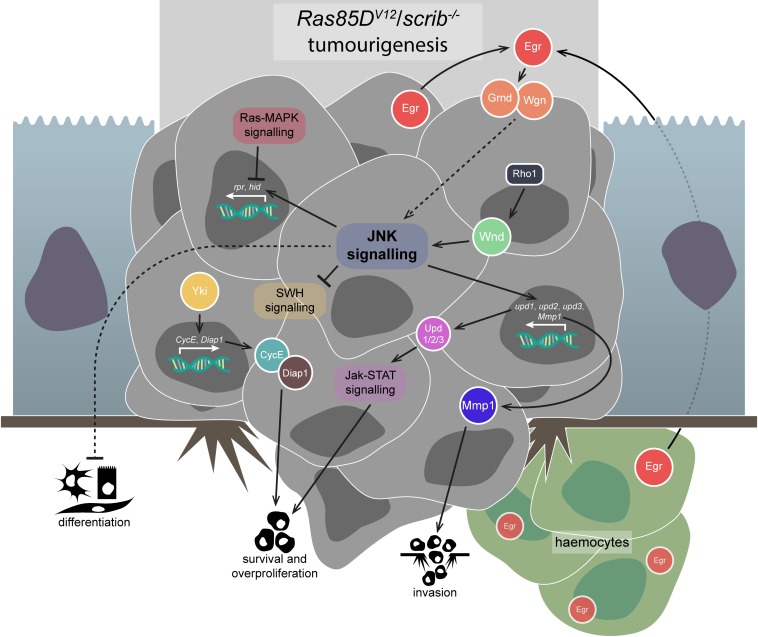
Pro-tumourigenic JNK signalling. JNK signalling in the face of apoptosis-suppressing signals, like that which occur via Ras-MAPK signalling in *Ras85D*^*V12*^/*scrib*^–/–^ tumours, is co-opted to promote several tumourigenic behaviours. JNK signalling in these tumours is activated via some combination of Egr-mediated TNFR activation and Rho1-Wnd signalling – the proportions of each are not fully understood, but TNF signalling has been shown to be dispensable. JNK signalling suppresses differentiation in *Ras85D*^*V12*^/*scrib*^–/–^ tumours, but the precise mechanism of this interaction is unclear. JNK signalling also appears to suppress SWH signalling, allowing Yki to promote the survival and overproliferation of the tumourous cells. Jak-STAT signalling, initiated by the Upd-family ligands whose expression is promoted by JNK signalling, also contributes to tumourigenic survival and overproliferation. Lastly, JNK signalling promotes invasiveness of the tumour cells and basement membrane degradation by upregulating proteins such as Mmp1. Gene and protein name abbreviations used in the diagram are as follows: Eiger (Egr), Grindelwald (Grnd), Wengen (Wgn), Wallenda (Wnd), *unpaired 1* (*upd1*), *unpaired 2* (*upd2*), *unpaired 3* (*upd3*), *Matrix metalloproteinase 1* (*Mmp1*), Yorkie (Yki), *Cyclin E* (*CycE*), *Death-associated inhibitor of apoptosis 1* (*Diap1*), *reaper* (*rpr*), *head involution defective* (*hid*).

Pro-tumourigenic JNK signalling in *Drosophila* was discovered during the study of cooperative tumourigenesis. Cancer is a multi-step process, and cooperative tumourigenesis is the phenomenon by which different genetic lesions in a cell, or in different cells, can cooperate to drive the initiation and progression of cancer. In *Drosophila*, cooperative tumourigenesis was discovered by one group of researchers looking at the consequences of introducing oncogenic mutations into *scrib*^–/–^ clones (Brumby and Richardson, 2003), and simultaneously by another group of researchers screening for mutations that cooperate with oncogenic mutations to produce metastatic tumours (Pagliarini and Xu, 2003). Coming from opposite directions, both groups identified that expressing an activated form of *Ras oncogene at 85D* (*Ras85D* – the most commonly used activated form is often referred to as *Ras*^*V12*^, but hereafter is referred to as *Ras85D*^*V12*^) cooperated with mutations in cell polarity regulator genes such as *scrib* to produce overgrown and invasive tumours in eye-antennal imaginal discs (Figure 3; Brumby and Richardson, 2003; Pagliarini and Xu, 2003). Ras85D is a GTPase, and canonically acts via the “Ras-MAPK” signalling pathway to effect gene transcription.

These initial studies did not determine a role for JNK signalling in *Ras85D*^*V12*^/*scrib*^–/–^ tumours, but it was clear that JNK-mediated apoptosis must be blocked in some way (Brumby and Richardson, 2005). Surprisingly, the JNK signalling pathway was in fact strongly upregulated in *Ras85D*^*V12*^/*scrib*^–/–^ tumours (and was not upregulated in the benign tumours formed after expression of *Ras85D*^*V12*^ in isolation) (Igaki et al., 2006; Uhlirova and Bohmann, 2006). Indeed, JNK signalling was necessary (and sufficient when induced via activated Hep, but insufficient when induced via Egr overexpression) for *Ras85D*^*V12*^/*scrib*^–/–^ tumour invasiveness, which was further demonstrated to be due to the JNK-induced transcription of *Matrix metalloproteinase 1* (*Mmp1*) (Figure 3; Igaki et al., 2006; Uhlirova and Bohmann, 2006). Mmp1 is from a family of genes strongly linked to cell motility, and is necessary for basement membrane degradation and invasive behaviours by *Ras85D*^*V12*^/*scrib*^–/–^ tumours (Srivastava et al., 2007). Other JNK signalling targets that are thought to contribute to invasive behaviours include the actin cross-linker *cher* (Pastor-Pareja et al., 2004; Külshammer and Uhlirova, 2013) and the integrin-associated scaffolding protein Paxillin (Huang et al., 2003; Llense and Martín-Blanco, 2008; Leong et al., 2009).

It was therefore thought that JNK signalling was switched from pro-apoptotic to pro-growth/proliferation in the face of *Ras85D*^*V12*^, a role that was known from experiments in undead cells – when apoptosis is triggered, but caspase activity is prevented via expression of the effector caspase inhibitor p35, cells are referred to as “undead” and undergo behaviours associated with cell death, but remain in the tissue and can induce non-autonomous effects (see also section “Non-autonomous effects of JNK signalling”) (reviewed in Martín et al., 2009). In *Drosophila* wing imaginal disc cells, undead cells induce JNK-dependent overproliferation in their *wildtype* neighbours, which reflects this observed role reversal (Pérez-Garijo et al., 2004; Ryoo et al., 2004). Similarly, JNK signalling resulting from non-apoptotic levels of caspase activation in undead cells also autonomously promotes invasiveness via *Mmp1* upregulation (Rudrapatna et al., 2013).

The aforementioned studies demonstrate that the malignancy of *Ras85D*^*V12*^/*scrib*^–/–^ tumours depends on JNK signalling, and the downstream effectors of that JNK signal have recently been identified. Three key TFs act downstream of JNK signalling in *Ras85D*^*V12*^/*scrib*^–/–^ tumours – Kay (a.k.a. Fos), Ftz TF 1 (Ftz-f1), and Ets at 21C (Ets21C) (Külshammer et al., 2015). Similarly, another study demonstrated that a majority of the phenotypes seen in *Ras85D*^*V12*^/*scrib*^–/–^ tumours can be traced back to a network of around 10 interconnected TFs that act downstream of JNK, SWH, and Jak-STAT signalling (Atkins et al., 2016), with Jak-STAT signalling being a key contributor to *Ras85D*^*V12*^/*scrib*^–/–^ tumour overgrowth (Figure 3; Wu et al., 2010; Atkins et al., 2016). Regardless, it was shown that Kay was solely responsible for JNK-related differentiation defects and *Mmp1* upregulation, but both Kay and Ftz-f1 are necessary for tumour invasiveness, and Ets21C overexpression can cooperate with Ras85D^*V12*^ to produce invasive (but non-overgrowing) clones (Külshammer et al., 2015).

Jun N-terminal kinase signalling inhibits differentiation of *Ras85D*^*V12*^/*scrib*^–/–^ tumour cells via an unclear mechanism (Figure 3), but it can be observed in the eye imaginal disc tissue due to decreased expression of embryonic lethal abnormal vision (Elav), a marker of photoreceptor cell differentiation, which is restored upon JNK signalling inhibition (Leong et al., 2009). When cooperative tumourigenesis between *Ras85D*^*V12*^ and *scrib*^–/–^ was first identified, it was also demonstrated that an activated form of Notch (N – the activated form is commonly and hereafter referred to as N^*ACT*^) also cooperated with *scrib*^–/–^ to induce tumourigenesis (Brumby and Richardson, 2003). While invasiveness of *N*^*ACT*^/*scrib*^–/–^ tumours is driven by JNK signalling, differentiation suppression is not, as blocking JNK did not rescue differentiation as indicated by Elav expression (Leong et al., 2009). However, blocking aPKC and JNK simultaneously was able to completely rescue the differentiation defects in, and the overgrowth and invasion phenotypes of, *N*^*ACT*^/*scrib*^–/–^ tumours (Leong et al., 2009). Interestingly, it was recently found that *N*^*ACT*^ can also cooperate with *l(2)gl* mutation to produce tumours, which also have their invasiveness driven by JNK-induced Mmp1 activity (Paul et al., 2018). Similarly, ectopic JNK signalling also contributes to tumourigenesis driven by *Ras85D*^*V12*^ expression and *l(2)gl* mutation – *Ras85D*^*V12*^/*l(2)gl^–/–^* tumours upregulate JNK signalling, which is thought to proceed via Src oncogene at 42A (Src42A), Ubiquitin-conjugating enzyme variant 1A (Uev1A), and the E2 ubiquitin ligase, Bendless (Ben) (Ma et al., 2013a, b). Uev1A and Ben play a highly conserved role in regulating the DNA damage response in cells via their role in K63-linked polyubiquitination, but whether such activity is required in JNK signalling remains to be determined (Bai et al., 2018). Activation of the Wingless (Wg, orthologue of the human *WNT* family) signalling pathway by JNK signalling is also thought to drive invasiveness via upregulation of *Mmp1* expression and activity in *Ras85D*^*V12*^/*l(2)gl^–/–^* tumours (Zhang et al., 2019). Indeed, direct activation of JNK signalling together with *Ras85D*^*V12*^ expression is sufficient for neoplastic tumourigenesis (Brumby et al., 2011).

While there are clearly differences between *Ras85D*^*V12*^/*scrib*^–/–^ and *N*^*ACT*^/*scrib*^–/–^ tumours, research has also uncovered many genetic similarities between them. Microarray data from *Ras85D*^*V12*^/*scrib*^–/–^ and *N*^*ACT*^/*scrib*^–/–^ tumours has identified just over 500 genes that were similarly misregulated between the two tumours, as well as 103 genes that were specifically responsive to JNK signalling shared between them (Doggett et al., 2015). Four of those genes were BTB-zinc finger TFs, and one of those was *chronologically inappropriate morphogenesis* (*chinmo*), which was shown to be capable of cooperating with both *Ras85D*^*V12*^ and *N*^*ACT*^ to drive tumourigenesis, even if JNK signalling was blocked (Doggett et al., 2015). A similar role was identified for the BTB-zinc finger TF *fruitless*, while another BTB-zinc finger TF, *abrupt*, was able to compensate for *chinmo* removal in driving tumourigenesis of *scrib^–/–^ /Ras85D^*V12*^* clones (Doggett et al., 2015). This indicates that these BTB-zinc finger TFs are important transcriptional targets of JNK signalling in cooperative tumourigenesis.

As with JNK in its anti-tumourigenic role, questions exist regarding the source of pro-tumourigenic JNK signalling. Egr is suspected, and haemocytes appear to be attracted to tumourous tissue just as they are attracted to pre-tumourigenic tissue (Figure 3), though not necessarily by the same mechanism – *Ras85D*^*V12*^/*scrib*^–/–^ tumours, in their undead-like state, have been shown to co-opt the activity of caspases to generate reactive oxygen species (ROS), which can attract haemocytes, which then activate JNK signalling and caspases in the tumourigenic cells, forming a feedback loop (Pérez et al., 2017). It is also thought that the increased JNK signalling in *Ras85D*^*V12*^/*scrib*^–/–^ tumours may increase haemocyte proliferation via JNK-dependent upregulation of the *unpaired* (*upd1*, *upd2*, and *upd3*) family of genes (behaviourally similar to mammalian IL-6), which act as ligands for the proliferation-promoting Jak-STAT signalling pathway (Pastor-Pareja et al., 2008; Cordero et al., 2010; Wu et al., 2010; Bunker et al., 2015). Regardless, haemocytes produce Egr when associated with pre-tumourigenic and tumourigenic tissue, but while Egr activates apoptosis promoting TNF-JNK signalling in pre-tumourigenic tissue, it is thought to promote tumour growth in *Ras85D*^*V12*^/*scrib*^–/–^ tumourigenic tissue, as well as invasive capacity via TNF-JNK-mediated *Mmp1* upregulation (Cordero et al., 2010). However, there is debate regarding the importance of Egr in JNK activation in polarity-impaired tumourigenesis. It has recently been shown that, in two different JNK-driven tumourigenesis models in the eye-antennal imaginal discs – tumourigenesis induced via polarity-impairment (*Ras85D*^*V12*^/*scrib*^–/–^) or via chromosomal instability (CIN) – JNK signalling initiation primarily derives from the tumourous epithelia itself, rather than recruited haemocytes or mesenchymal myoblasts (Muzzopappa et al., 2017). Furthermore, it was found that *egr* and *grnd* were dispensable in the process – instead, JNK signalling derived from signalling through the JNKKKs Wnd and Ask1 in polarity-impairment- and CIN-induced tumourigenesis, respectively (Figure 3; Muzzopappa et al., 2017). The authors reasoned this was due to CIN-induced tumourigenesis producing ROS, to which Ask1 is sensitive (Sekine et al., 2012), while Wnd mediates JNK signalling in response to polarity-impairment (see also section “Upstream regulation of JNK signalling”) (Ma et al., 2016; Muzzopappa et al., 2017).

We have highlighted how anti-tumourigenic JNK signalling is thought to be partly responsible for blocking Yki activation in pre-tumourigenic *scrib*^–/–^ clones (Doggett et al., 2011; Chen et al., 2012). However, it is currently unclear whether co-opting JNK signalling into being pro-tumourigenic in *Ras85D*^*V12*^/*scrib*^–/–^ tumours alters its effect on SWH signalling. In *Ras85D*^*V12*^/*scrib*^–/–^ tumourigenesis, SWH signalling is impaired, and Yki is active and contributes to the observed neoplastic overgrowth, but not invasion (Figure 3; Doggett et al., 2011). Conversely, it has been demonstrated that Yki-driven overgrowth in wing and eye-antennal imaginal discs is suppressed via JNK-mediated Wts activity (Enomoto et al., 2015). However, when *Ras85D*^*V12*^ is coupled with active JNK signalling (via *egr* overexpression) tumourigenesis occurs similar to that driven by *Ras85D*^*V12*^/*scrib*^–/–^, and the combination of Ras-MAPK and JNK signalling leads to Yki activation via the accumulation of F-actin, dependent on the actin regulators Jub, Diaphanous (Dia), and Rac1, as well as inactivation of Wts (Enomoto et al., 2015). These data suggest SWH signalling modulation and Yki upregulation contribute to *Ras85D*^*V12*^/*scrib*^–/–^ tumourigenesis, but more research is needed to understand how JNK signalling interacts with SWH signalling and Yki during the process.

As briefly discussed, tumourigenesis can be modelled in *Drosophila* by more mechanisms than just oncogene activation coupled with polarity-impairment (as per *Ras85D*^*V12*^/*scrib*^–/–^), and many of these alternative mechanisms are also reliant on JNK signalling. However, while activated Ras85D is known to block apoptosis (Bergmann et al., 1998; Kurada and White, 1998) and thus co-opt JNK signalling into promoting tissue growth (Figure 3), in other cases of cooperative tumourigenesis it is not clear how the apoptosis promoting role of JNK is halted. Examples of both such JNK-driven tumour types have recently been described elsewhere (reviewed in Richardson and Portela, 2018). We list some newly identified examples of JNK-driven tumourigenesis in various *Drosophila* tissues below.

(1) Aneuploid cells formed via CIN undergo tumourigenesis via JNK signalling activation, with the delamination and invasive behaviour of the cells driven by JNK targets promoting misregulation of the actin-myosin cytoskeleton (Benhra et al., 2018).
(2) *Src oncogene at 64B* (*Src64B*) and *Src42A* are capable of inducing tumourigenesis in eye-antennal imaginal discs when overexpressed by cooperating with *Ras85D*^*V12*^ – JNK signalling in these tumours is necessary for their neoplastic overgrowth and invasion, and the Raf-MAPK and Phosphoinositide 3-kinase (PI3K) signalling pathways act downstream of Ras85D^*V12*^ to facilitate this cooperation (Poon et al., 2018). These findings are consistent with a previous study that demonstrated the upregulation of Src family genes alone is capable of activating JNK signalling in the wing imaginal disc, and promotes invasion via actin cytoskeleton remodelling (Rudrapatna et al., 2014).
(3) A *Drosophila* glioblastoma, complete with tumour cell-interconnecting microtubules (TMs), is driven by constitutively active EGFR and PI3K signalling in glial cells (Read et al., 2009; Portela et al., 2019). Wg signalling is activated in the glioma cell TMs due to “vampirisation” of the ligand from the surrounding *wildtype* neurons and drives tumour progression (Portela et al., 2019). Furthermore, TNF-JNK-Mmp1/2 signalling, acting via Grnd, is also upregulated in the TMs, is necessary for the “vampirisation” process, and forms a positive feedback loop with Wg signalling by promoting TM formation (Portela et al., 2019).
(4) Epigenetic silencers of the Polycomb Group (PcG) can cause tumourigenesis to occur if mutated (Beira et al., 2018). One PcG family member is *polyhomeotic*, the clonal mutants of which upregulate JNK signalling via Egr and Grnd (as well as Notch and Jak-STAT signalling), and promote neoplastic overgrowth, invasion, and polarity loss (Beira et al., 2018).
(5) Genes involved with the endocytic process, such as *Rab5*, *Syntaxin 7* (*Syx7*, a.k.a *avalanche*), *Tumour susceptibility gene 101* (*TSG101*, a.k.a. *erupted*), and various Vacuolar protein sorting family genes, represent another relatively well-studied class of nTSGs. In tissues predominantly mutant for these genes, JNK signalling is upregulated, and shares tumourigenesis-promoting roles with Jak-STAT signalling (Woodfield et al., 2013). Similar phenomena are observed upon overexpression of Vacuolar H+ ATPase 44kD subunit (Vha44), the C-subunit of V-ATPase, which is involved in the acidification of endosomes, and also leads to JNK-dependent tumourigenesis (Petzoldt et al., 2013). More recently, it has been shown that endocytic nTSG clones generated in eye-antennal imaginal discs also have some degree of polyploidy due to JNK and Yki coactivation, with JNK signalling downregulating the G2-M phase cell cycle regulator *Cyclin B* (*CycB*) and Yki upregulating *Diap1* to promote polyploidy-inducing endoreplication (Cong et al., 2018). Interestingly, it was shown that polyploid cells also form in *Ras85D*^*V12*^/*scrib*^–/–^ tumours due to *CycB* downregulation, and blocking their formation inhibits the invasive behaviours of these tumours (Cong et al., 2018).
(6) Jun N-terminal kinase signalling can be important in tumourigenesis at transition zones – where different epithelial cell populations meet, which are often hotspots for tumourigenesis (reviewed in Tamori and Deng, 2017). One such zone occurs in the *Drosophila* larvae at a site where polyploid salivary gland cells meet the diploid imaginal ring cells, where tumourigenesis occurs after transient whole animal *N*^*ACT*^ expression due to upregulation of TNF-JNK and Jak-STAT signalling (Yang et al., 2019).
(7) Overexpression of Canoe (Cno, an adherens junction scaffold protein) in the *patched* (*ptc*) expression domain in wing imaginal discs conversely promotes both overproliferation and ectopic cell death, as well as cell migration/invasion (Ma et al., 2019). While JNK signalling was upregulated in the Cno-expressing cells, moderate inhibition of JNK signalling was able to block cell death and promote massive tissue overgrowth, while strong JNK inhibition led to only partial overgrowth (Ma et al., 2019), indicating JNK signalling levels are balancing pro- and anti-tumourigenic roles in this model.

In summary, the second face of JNK signalling is as a powerful driver of tumourigenesis. Pro-proliferation and survival functionalities are co-opted by apoptosis suppression signals such as Ras-MAPK signalling, and JNKs regulation of cell movement and migration is converted into promoting invasion and metastasis. Modulation of SWH signalling is thought to be involved with the pro-tumourigenic roles of JNK, but more research is needed to fully clarify this signalling cross-talk. JNK signalling in *Drosophila* is therefore a powerful pro-tumourigenic force, but context is key.

## Non-Autonomous Effects of JNK Signalling

Most of our previous discussions regarding the effects of JNK signalling dealt with autonomous induction and action of the pathway, however, non-autonomous effects of JNK signalling have been identified. One of the earliest explorations of non-autonomous JNK signalling effects came from examinations of cooperative tumours generated via *scrib* mutation and *Raf oncogene* (*Raf*) activation (using a *Raf* gain-of-function allele (*Raf*^*GOF*^)) (Uhlirova et al., 2005). These *Raf*^*GOF*^/*scrib*^–/–^ tumours are indistinguishable from *Ras85D*^*V12*^/*scrib*^–/–^ tumours. Upregulating JNK signalling in *Raf*^*GOF*^/*scrib*^–/–^ tumours via *hep*^*ACT*^ expression led to a reduction in the size of eye-antennal imaginal discs, but adult eyes increased in size, and in both cases the GFP-positive cells (where the different transgenes were clonally expressed) were eliminated, suggesting a non-autonomous effect on the growth of the surrounding *wildtype* tissue (Uhlirova et al., 2005). The researchers suggested that the addition of *hep*^*ACT*^ overcomes the apoptosis inhibition of *Raf*^*GOF*^, and prompts compensatory proliferation from the *wildtype* cells, but the secretion of cytokines by the *Raf*^*GOF*^/*scrib*^–/–^/*hep*^*ACT*^ cells leads to the malformation observed (Uhlirova et al., 2005).

**FIGURE 4:**
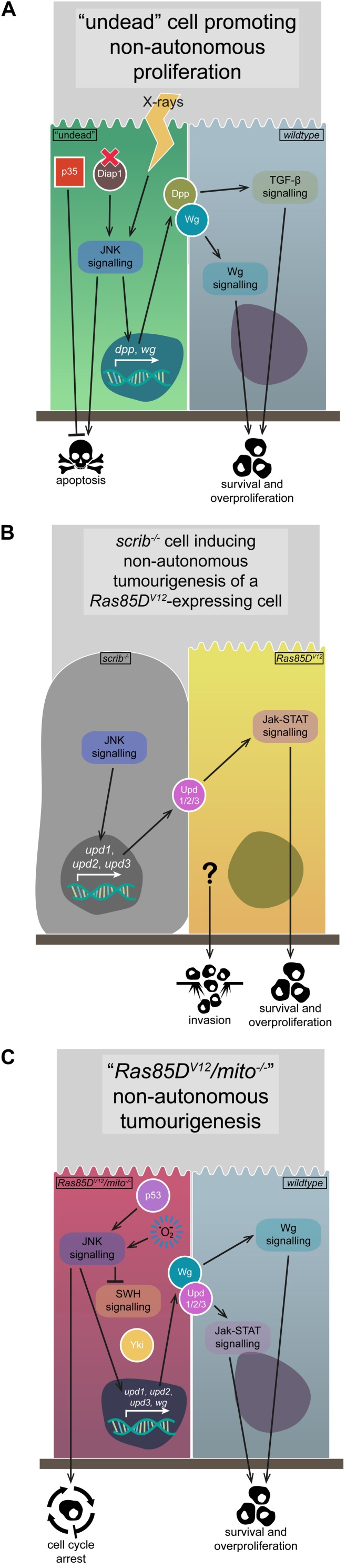
Non-autonomous JNK signalling. The activation of JNK signalling in one group of cells can have non-autonomous effects on the growth and proliferation of their neighbours, due to the upregulation of various signalling pathway initiators. **(A)** The generation of “undead” cells via the upregulation of p35 while simultaneously inducing apoptosis via *Diap1* mutation or X-ray application leads to JNK signalling activation, the expression and secretion of Wg and Dpp ligands, and the survival and overproliferation of neighbouring cells due to Wg and TGF-β signalling pathway activation. **(B)** The upregulation of JNK signalling that occurs in *scrib*^–/–^ cells leads to expression and secretion of the Upd-family ligands, which can activate Jak-STAT signalling in neighbouring *Ras85D*^*V12*^-expressing tumourigenic cells, leading to their survival and overproliferation, and possibly their invasiveness. **(C)** Cells expressing *Ras85D*^*V12*^ coupled with mitochondrial gene mutations upregulate JNK signalling, due to p53 and ROS activity. Said JNK signalling downregulates SWH signalling, derepressing Yki, and also upregulates expression and secretion of Wg and the Upd-family ligands, activating Jak-STAT and Wg signalling in neighbouring cells and promoting their survival and overproliferation. Gene and protein name abbreviations used in the diagram are as follows: Death-associated inhibitor of apoptosis 1 (Diap1), *decapentaplegic* (d*pp*), *wingless* (*wg*), *unpaired 1* (*upd1*), *unpaired 2* (*upd2*), *unpaired 3* (*upd3*), Yorkie (Yki).

Compensatory proliferation is one of the key mechanisms through which non-autonomous JNK signalling is realised, and it is usually effected via secretable JNK targets that induce proliferation, such as Wg or the Upd family ligands (Uhlirova et al., 2005; Pastor-Pareja et al., 2008; Sun and Irvine, 2011). While a healthy level of compensatory proliferation maintains tissue homeostasis in the face of wounding or cell competition, the process can be corrupted if apoptosis is induced (e.g., by *Diap1* mutation or X-ray exposure) but the pathway is blocked by *p35* expression – these undead cells upregulate JNK signalling, which promotes the expression and secretion of Wg and Decapentaplegic (Dpp), ligands that then promote the non-autonomous overproliferation and overgrowth of neighbouring cells (Figure 4A; Pérez-Garijo et al., 2004; Ryoo et al., 2004). Interestingly, TNF-JNK signalling in undead cells can also, conversely, trigger non-autonomous apoptosis, with cell death being induced in different wing imaginal disc compartments, a process the researchers termed “apoptosis-induced apoptosis” (Pérez-Garijo et al., 2013).

Perhaps unsurprisingly, non-autonomous effects of JNK signalling can be tumourigenic, such as the phenomenon of interclonal cooperation, where *scrib*^–/–^ (or *l(2)gl^–/–^*) clones adjacent to *Ras85D*^*V12*^-expressing clones (*Ras85D*^*V12*^//*scrib*^–/–^) cooperate to induce tumourigenesis of the *Ras85D*^*V12*^ cells – these tumours appear functionally identical to those where *scrib* is mutated and *Ras85D*^*V12*^ is expressed in the same cells (*Ras85D*^*V12*^/*scrib*^–/–^) (Figure 4B; Wu et al., 2010). Researchers found that the interclonal cooperation was due to the secretion of the Upd family ligands, which activate Jak-STAT signalling and are targets of JNK-mediated transcription in *scrib*^–/–^ cells (Figure 4B; Wu et al., 2010; Bunker et al., 2015). Indeed, co-expression of *upd1*, *upd2*, or *upd3* and *Ras85D*^*V12*^ replicated the interclonal cooperation phenotype, and inhibiting JNK signalling rescued *Ras85D*^*V12*^//*scrib*^–/–^ tumourigenesis, but not *Ras85D*^*V12*^/*upd1/2/3* tumourigenesis (Figure 4B; Wu et al., 2010). In this example of interclonal cooperation, the *scrib*^–/–^ cells upregulate JNK signalling and, while they are eventually eliminated, the JNK signal non-autonomously allows for the tumourigenic overgrowth of the *Ras85D*^*V12*^-expressing cells (Wu et al., 2010).

Another example of non-autonomous JNK signalling activity is the case of *Ras85D*^*V12*^ cooperation with mutated genes from the mitochondrial respiratory system, including *NADH dehydrogenase (ubiquinone) PDSW subunit*, *mitochondrial ribosomal protein L4*, and *Cytochrome c oxidase subunit 5A* – their cooperation induces overgrowth in *wildtype* neighbours, but not in the mutated cells (Ohsawa et al., 2012). ROS production in the *Ras85D*^*V12*^/mitochondrial gene mutant clones (*Ras85D^*V12*^/mito^–/–^*) promotes ectopic JNK signalling, which contributes to SWH downregulation, Yki upregulation, and the transcription of *wg* and *upd1*/*upd2*/*upd3* (Ohsawa et al., 2012). The expression and secretion of Wg and Upd1/Upd2/Upd3 acts on the surrounding *wildtype* cells to promote their proliferation (Figure 4C), but if the surrounding cells overexpress *Ras85D*^*V12*^, they develop into neoplastic invasive tumours (Ohsawa et al., 2012). Whether tumourigenic or not, the Upd gene family being a transcriptional target of JNK signalling is a common theme in how non-autonomous JNK signalling is effected – this is also seen in clones mutant for the early endosomal regulatory gene *Rab5*, where the concomitant disruptions to endocytic processes lead to upregulated TNF-JNK and Ras-MAPK signalling, Yki activation, and Upd ligand expression and secretion to drive overgrowth of surrounding tissue (Takino et al., 2014). Research using the same model system (*Ras85D^*V12*^/mito^–/–^* clones) further dissected how JNK signalling was regulated and acted. It was shown that *Ras85D^*V12*^/mito^–/–^* clones displayed phenotypes associated with cellular senescence – their cell cycle was arrested in G1, they upregulated various senescence-associated markers, the individual cells were overgrown, and they displayed a senescence-associated secretory phenotype (SASP) (Figure 4C; Nakamura et al., 2014). In these cells, ROS production and p53 upregulation contribute to the activation of JNK signalling, which then induces Upd1/Upd2/Upd3 expression and secretion, leading to non-autonomous tissue growth effects (Figure 4C; Nakamura et al., 2014). A somewhat related role for JNK signalling was recently observed for cells upon wounding in wing imaginal discs – wounding induced JNK signalling, and cells became transiently “stalled” in the cell cycle at G2 phase (or near-permanently “arrested” in G2 phase after JNK induction via *egr* overexpression in the *rotund* expression domain) (Cosolo et al., 2019). Researchers found that JNK signalling induces G2 phase stalling/arrest via downregulating the activity of Stg (an inducer of mitosis) and, furthermore, that cells with this G2 profile were protected from JNK-mediated apoptosis (Cosolo et al., 2019). It was also shown that the G2-biased profile of clones mutant for *wts* or *dlg1* was due to JNK signalling (Cosolo et al., 2019). Interestingly, the *wildtype* tissue adjacent to these mutant clones overgrows, and was suggested by researchers to be another example of JNK-induced non-autonomous tissue growth (Cosolo et al., 2019). Similar cell cycle arrest has also been observed in wholly *scrib*^–/–^ wing imaginal disc tumours – strong JNK signalling in periphery cells early in tumourigenesis induces G2/M phase arrest, but JNK signalling decreases over time in these cells and, together with a concomitant increase in Ras-MAPK signalling, the cell cycle arrest ceases and the tumours overgrow (Ji et al., 2019).

One final example of JNK signalling-induced non-autonomous effects concerns imaginal disc clones overexpressing *Src64B* that are eliminated via cell competition, but cause the overgrowth of their *wildtype* neighbours (Enomoto and Igaki, 2013). Clonal *Src64B*-overexpression activates JNK signalling, in part via the induction of F-actin accumulation (Enomoto and Igaki, 2013) – further links have been drawn between the actin cytoskeleton and JNK signalling activation, as Src genes have also been shown to promote JNK signalling via Rho1-induced actin remodelling (Rudrapatna et al., 2014). Regardless, the F-actin accumulation also promotes Yki activation in the *Src64B*-overexpressing clones – though their overgrowth is opposed by the upregulated JNK signal, the Yki signal propagates to the *wildtype* neighbours in a JNK-dependent manner, and there upregulates Yki and promotes non-autonomous overgrowth (Enomoto and Igaki, 2013). Interestingly, however, if JNK signalling is inhibited in the *Src64B*-overexpressing clones, Yki activity promotes the tumourigenic overgrowth of those cells instead (Enomoto and Igaki, 2013).

Non-autonomous JNK signalling is necessary for the maintenance of tissue homeostasis, regulating as it does the process of compensatory proliferation after cell competition or wounding events. However, the above examples also show that JNK signalling is two-faced, and can be co-opted to effect non-autonomous tumourigenesis, inducing or enhancing the overgrowth and invasion of otherwise benign cells.

## Upstream Regulation of JNK Signalling

The activation of JNK signalling is complex. While the kinase core remains largely the same, upstream activation contexts can vary wildly. Most of the cases discussed so far (where it has been examined) are thought to have utilised the TNF-JNK signalling pathway in various ways, but clearly this is not the only way JNK can be activated. In this section, we will discuss some of the more unique ways in which JNK signalling can be activated when acting in a pro- or anti-tumourigenic fashion.

### Regulating JNK Signalling in Development and Tissue Homeostasis

**FIGURE 5:**
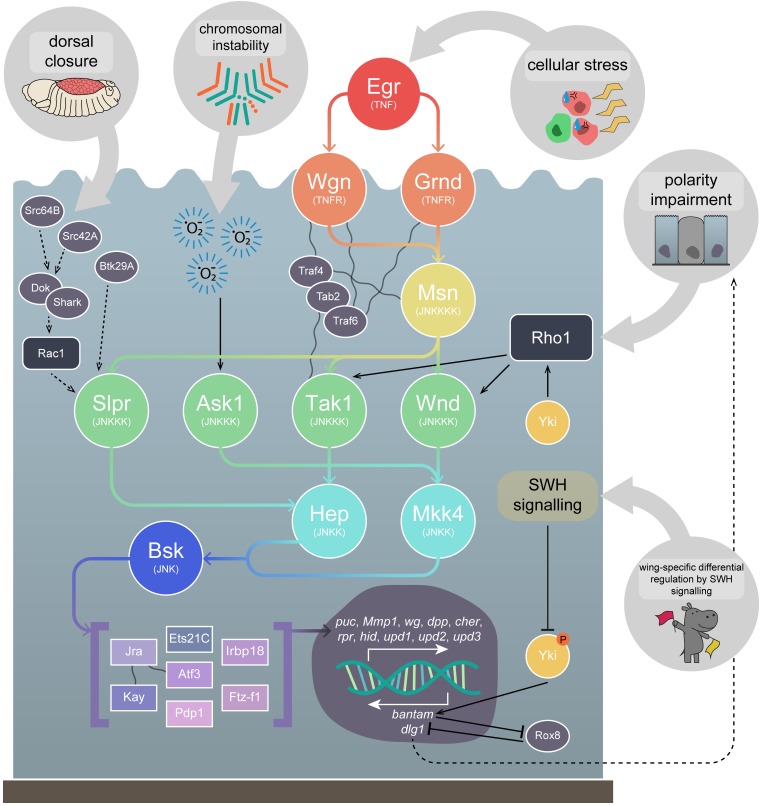
Upstream regulation of JNK signalling. JNK signalling can be activated by a variety of different upstream mechanisms. JNK signalling mediates cell morphology changes, such as those during embryonic dorsal closure, where it is thought to act via Src42A and Src64B (and/or Btk29A) to activate the kinase Shark and its adapter, Dok. This signalling may in turn activate Rac1 and promote JNK signalling via Slpr. In cells with chromosomal instability, accumulated ROS promote Ask1 signalling, stimulating the JNK signalling pathway to promote apoptosis. Arguably, the best understood pathway is the TNF-JNK signalling pathway, which is generally considered to be activated as a response to cellular stresses. Egr, the *Drosophila* TNF, binds to Grnd or Wgn (TNFRs), which activate Msn (JNKKKK) and Tak1 (JNKKK), via the adapter proteins Traf4, Tab2, and Traf6. JNK signalling is activated in polarity-impaired cells, which is thought to occur via the stimulation of Wnd by the actin cytoskeleton regulator Rho1, although TNF signalling contributes to amplify JNK activity. Lastly, in *Drosophila* wing imaginal discs, differential JNK signalling regulation by the SWH signalling pathway has been observed. While non-active SWH signalling allows Yki to promote JNK activation via Rho1, activated SWH signalling suppresses Yki activity, preventing *bantam* transcription, a miRNA that suppresses Rox8, which acts as a positive regulator of JNK signalling, possibly by downregulating *dlg1*. Msn is thought to be capable of activating Tak1, Wnd, and Slpr, but has not yet been shown to activate Ask1. Tak1, Wnd, and Ask1 are then thought to be capable of activating both the JNKKs, Hep and Mkk4, while Slpr has only yet been shown to act via Hep. Both Hep and Mkk4 can activate the sole *Drosophila* JNK, Bsk, which positively regulates a number of TFs, including the well known Jra and Kay. These TFs promote transcription of a number of important genes, including the apoptosis promoters *hid* and *rpr*, the Jak-STAT ligands *upd1*, *upd2*, and *upd3*, the invasion promoter *Mmp1*, and the negative JNK regulator *puc*. Dotted lines represent uncertain interactions. Wavy lines represent known physical interactions between core pathway members and (their adapters. Gene and protein name abbreviations used in the diagram are as follows: Src oncogene at 64B (Src64B), Src oncogene at 42A (Src42A), Btk family kinase at 29A (Btk29A), Downstream of kinase (Dok), SH2 ankyrin repeat kinase (Shark), Eiger (Egr), Grindelwald (Grnd), Wengen (Wgn), Misshapen (Msn), TNF-receptor-associated factor 4 (Traf4), TAK1-associated binding protein 2 (Tab2), TNF-receptor-associated factor 6 (Traf6), Slipper (Slpr), Wallenda (Wnd), TGF-β activated kinase 1 (Tak1), Apoptotic signal-regulating kinase 1 (Ask1), Hemipterous (Hep), MAP kinase kinase 4 (Mkk4), Basket (Bsk), Jun-related antigen (Jra), Kayak (Kay), Ets at 21C (Ets21C), Activating transcription factor 3 (Atf3), PAR-domain protein 1 (Pdp1), Inverted repeat binding protein 18 kDa (Irbp18), Ftz transcription factor 1 (Ftz-f1), puckered (puc), *Matrix metalloproteinase 1* (*Mmp1*), *wingless* (*wg*), *decapentaplegic* (*dpp*), *cheerio* (*cher*), *reaper* (*rpr*), *head involution defective* (*hid*), *unpaired 1* (*upd1*), *unpaired 2* (*upd2*), *unpaired 3* (*upd3*), Yorkie (Yki), *discs large 1* (*dlg1*).)

Besides acting as a pro-apoptotic signal, JNK signalling is arguably best understood as a regulator of cell morphology and migration in a variety of developmental contexts (reviewed in Harden, 2002; Ríos-Barrera and Riesgo-Escovar, 2013). During *Drosophila* embryogenesis, JNK signalling plays a critical role during the epithelial sheet migration process of dorsal closure, and similarly in thoracic closure during pupariation (Figure 5). Activation of JNK signalling during thoracic closure is mediated by Pvr signalling via Crk oncogene (Crk), Ced-12, Mbc, and Rac1 (Ishimaru et al., 2004). As an aside, constitutively activated Pvr signalling is oncogenic, and in wing imaginal discs activates JNK signalling (alongside Ras-MAPK and PI3K signalling) to effect metabolic reprogramming of the tumour cells (Wang et al., 2016). Another process in which both Pvr and JNK signalling are involved is border cell migration (BCM), a process during oogenesis involving the movement of a cluster of “border cells” from the apical end of the egg chamber to the surface of the oocyte itself, and which is an established model of cell migration and invasion in *Drosophila* (reviewed in Montell et al., 2012). During BCM, JNK signalling regulates clustering and migratory behaviours of the border cells, and is thought to be activated by the GTPases Rho1 and Cdc42 (Mathieu et al., 2007; Llense and Martín-Blanco, 2008; Melani et al., 2008). It is further thought that JNK signalling contributes to the Pvr signalling-mediated guidance of the border cells, but this interaction is not fully understood (Llense and Martín-Blanco, 2008).

Returning to dorsal and thoracic closure, in both processes Rac1 is thought to activate JNK signalling via the JNKKK Slpr (Garlena et al., 2010), whereas in thoracic closure the JNKKK Wnd has been shown to be dispensable (Ma et al., 2015b). JNK signalling via Slpr during dorsal closure is believed to be initiated by the activity of the Src family proteins (and/or Btk family kinase at 29A (Btk29A)) and their downstream targets, SH2 ankyrin repeat kinase (Shark) and Downstream of kinase (Dok), and that Rac1 may act downstream of these molecules (reviewed in Ríos-Barrera and Riesgo-Escovar, 2013) – however, the upstream regulation of JNK signalling in these developmental contexts is not fully understood (Figure 5). The role of JNK signalling during dorsal closure has also been shown to rely on additional signalling pathways. Transforming growth factor-β (TGF-β) signalling, activated by Dpp, has been demonstrated as acting to suppress the pro-apoptotic activity of the JNK signalling pathway – while the JNK-induced AP-1 TF complex promotes *rpr* expression and apoptosis, the TGF-β-induced TF Schnurri (Shn) suppresses *rpr* expression (Beira et al., 2014). This is an elegant example of how JNK signals can be co-opted during developmental events, like in tumourigenesis, via the mechanism of apoptosis suppression.

It is believed that JNK signalling-mediated cell death during development generally proceeds via input from the TNF signalling pathway. However, research in this vein has largely examined how TNF-JNK signalling proceeds after induction, rather than how endogenous TNF-JNK signalling is regulated or initiated. In the *Drosophila* eye, JNK-mediated cell death induced by Egr expression acts predominantly via Tak1, though Wnd plays a small role, whereas JNK-mediated cell death induced by Rac1 expression acts predominantly via Wnd, but Tak1 is dispensable (Ma et al., 2015b). Furthermore, cell death (and invasion) within the *Drosophila* wing imaginal disc can be induced via *scrib* knockdown in the *ptc* expression domain – this was also suppressed by *wnd* knockdown, which appears to act via both Hep and Mkk4 in this context (Ma et al., 2015b). This study demonstrates the complex nature of JNK signalling regulation. Research suggests Rho1, like Rac1, can activate JNK signalling, but does not do so identically. As mentioned, it has been shown that Rho1 is likely responsible for JNK signalling upregulation in response to polarity-impairment in *Ras85D^*V12*^/scrib^–/–^* tumours (Muzzopappa et al., 2017). Furthermore, it has been shown that knockdown of *Rho1* (and *wnd*) suppressed *scrib* knockdown-induced cell death and invasion in the *ptc* expression domain in wing imaginal discs (Figure 5; Ma et al., 2016). Overexpression of *wnd* or *Rho1* in the *ptc* expression domain were found to promote epithelial to mesenchymal transition (EMT)-like invasive phenotypes and *Mmp1* upregulation (Figure 5; Ma et al., 2016). Knocking down *wnd* while overexpressing *grnd* (and *vice versa*) led to a rescue of the invasion phenotypes (though actin remodelling was not dependent on JNK signalling), suggesting some kind of feedback loop may occur (Ma et al., 2016). Furthermore, while *Rac1* overexpression in the *ptc* expression domain also led to JNK-dependent invasive behaviours, it was shown that *wnd* was dispensable (in contrast to the aforementioned necessity of *wnd* in Rac1-induced JNK-mediated cell death in the eye (Ma et al., 2015b)) and, curiously, that both *Rho1* and *Rac1* overexpression-induced phenotypes were rescued upon simultaneous disruption of Tak1 and Slpr (Ma et al., 2016).

Overexpression of *wnd* has also been shown to cooperate with *Ras85D*^*V12*^ to generate tumours where, similar to what is seen in *Ras85D*^*V12*^/*scrib*^–/–^ tumours, it induces JNK signalling to promote upregulation of the Wg signalling pathway, promoting cell proliferation (Ma et al., 2016). Various cell morphology regulators have also been identified as interacting with *Ras85D*^*V12*^ to promote both tumourigenesis and JNK signalling. These include the aforementioned Rho-family GTPases *Rho1* and *Rac1*, as well as their partner *Rho guanine nucleotide exchange factor 2* (*RhoGEF2*), which cooperate with *Ras85D*^*V12*^ to enhance adult eye overgrowth phenotypes when overexpressed, due to their role as positive regulators of JNK signalling (Brumby et al., 2011). Moreover, clonally, *Rac1*, *RhoGEF2*, or activated *Rho1* expression in isolation led to clones that were eliminated, but cooperated when expressed alongside *Ras85D*^*V12*^ to induce the formation of invasive tumours via activation of JNK signalling (Brumby et al., 2011). Specifically, RhoGEF2 and Rho1 act upstream of Rho kinase (Rok) and Spaghetti squash (Sqh, a.k.a Myosin II Regulatory Light Chain) to activate JNK signalling and cooperate with *Ras85D*^*V12*^ in tumourigenesis, but they also promote actin/myosin contractility and cell shape changes independently of JNK signalling (Khoo et al., 2013).

### TNF-JNK Signalling

TNF-JNK signalling has already been discussed in earlier sections, but a more detailed exploration is pertinent, as it is one of the best understood activation contexts of the JNK pathway. The TNF ligand of the pathway, Egr, was identified in 2002 as an orthologue of multiple members of the mammalian TNF gene family (Igaki et al., 2002; Moreno et al., 2002). Identification of the first *Drosophila* TNFR orthologue, Wgn, quickly followed (Kanda et al., 2002). It is thought that TNF-JNK signalling occurs primarily via interactions between activated TNFRs, the JNKKK kinase (JNKKKK) Misshapen (Msn), and the JNKKK Tak1, which are mediated by the adapter proteins TNF-receptor-associated factor 4 (Traf4), TNF-receptor-associated factor 6 (Traf6), and TAK1-associated binding protein 2 (Tab2) (Figure 5; reviewed in Igaki and Miura, 2014). The pathway then drives transcription via the canonical kinase core of Hep and Bsk, and TFs including the AP-1 complex members Kay and Jra – these effectors are, as we have discussed, primarily involved in mediating apoptosis as a response to cellular stresses, such as polarity-impairment, but can be co-opted into a role in tumourigenesis.

Recently, another *Drosophila* TNFR-encoding gene, *grnd*, was identified, and found to also play a role in the apoptosis of polarity-impaired cells, as well as in cooperative tumourigenesis (Figure 5; Andersen et al., 2015). It was found in a screen for genes necessary for neoplastic growth induced by knockdown of the endocytic gene *Syx7*, where most genes found to rescue tumourigenesis when disrupted were JNK pathway components (Andersen et al., 2015). Overexpression of *egr* in the adult eye results in cell death, which was rescued upon knockdown of *grnd*, but not of *wgn* (Andersen et al., 2015). It is thought that Grnd binds Egr, prevents its diffusion, and hence controls the autonomy of cell death – it was shown that wing imaginal disc clones overexpressing *egr* were eliminated via apoptosis, and co-expressing RNAi against *Tak1* prevented that autonomous cell death, but co-expressing RNAi against *grnd* prevented autonomous cell death while also promoting non-autonomous cell death (Andersen et al., 2015). Interestingly, proper identification of a classic *Drosophila* tumour suppressor gene, *lethal(2)tumorous imaginal discs*, as *ALG3, alpha-1,3- mannosyltransferase* (*Alg3*), has shed light on how Grnd might be regulated – *Alg3* mutants fail to glycosylate (and thus inactivate) Grnd, enabling persistent TNF-JNK signalling activation via Egr secreted by the fat body, which promotes JNK-mediated tissue overgrowth via SWH signalling inhibition and Yki activation (de Vreede et al., 2018). Furthermore, Grnd is likely to be involved in both polarity-impairment-induced cell competition and cooperative tumourigenesis – clones with *scrib* knockdown die, while those with knockdown of both *scrib* and *grnd* survive, and the invasiveness of *Ras85D*^*V12*^/*scrib*^–/–^ tumours is blocked via *grnd* knockdown, as is their overexpression of the JNK target, *Mmp1* (Andersen et al., 2015).

As we have discussed, there is some debate over how JNK signalling is activated in polarity-impaired cells – some studies implicate Egr as the key effector of the pathway (Igaki et al., 2009; Vidal, 2010), but others have suggested JNK signalling is initiated by direct activation of JNKKKs by molecules involved in cytoskeletal regulation, such as Rho1 (Ma et al., 2016; Muzzopappa et al., 2017). In this vein, one recent study has demonstrated a new method by which Egr can regulate polarity-impaired cell elimination. Knocking down *scrib* via RNAi in wing imaginal discs, within the *spalt major* expression domain, leads to large areas of polarity-impaired cells upregulating JNK signalling (Poernbacher and Vincent, 2018). Disrupting *egr* in those same cells was shown to only partially rescue the ectopic JNK signalling, while disrupting *grnd* or *adenosine receptor* (*AdoR*) completely rescued the ectopic JNK signalling (Poernbacher and Vincent, 2018). *AdoR* positively regulates JNK signalling when *scrib* is knocked down in various expression domains, and also in wholly *scrib*^–/–^ imaginal discs (Poernbacher and Vincent, 2018). Researchers found that the activation of JNK signalling was likely due to an increase in extracellular adenosine secreted by the polarity-impaired cells stimulating AdoR activity, which was then also necessary for *egr* transcript upregulation (Poernbacher and Vincent, 2018).

### Feedback Loops and ROS

An assortment of feedback loops involving JNK signalling have been identified in various contexts, allowing for persistent activation of the pathway. One of the key positive feedback loops identified occurs during apoptosis in response to stress, where death regulator Nedd2-like caspase (Dronc), an initiator caspase, is activated by and activates JNK signalling (Shlevkov and Morata, 2012; Rudrapatna et al., 2013). Other examples of positive feedback loops include when JNK signalling is activated due to signalling from the Src family members (Rudrapatna et al., 2014), and during *Ras85D^*V12*^/scrib^–/–^* tumourigenesis when haemocyte recruitment and ROS production promote JNK signalling (Pérez et al., 2017). Another feedback loop was recently identified that stresses the importance of JNK signalling activation in tumourigenesis progression, and also illustrates the stepwise process by which cooperative tumourigenesis can occur. Transient initiation of JNK signalling (via irradiation, *p53* expression, or activated *hep* (*hep*^*ACT*^) expression) in cells where apoptosis was functional led to activity of the pathway gradually ceasing (Pinal et al., 2018). Conversely, where apoptosis was blocked (via *Ras85D*^*V12*^ expression or apoptotic pathway component mutation) JNK signalling persisted, leading to overgrowth when *Ras85D*^*V12*^ was expressed and upregulation of growth-promoting JNK targets such as the Wg and Jak-STAT signalling pathway ligands (Pinal et al., 2018). Researchers identified that the transient JNK signalling-induced ROS production, and the sustained JNK signalling in apoptosis-deficient cells was dependent on ROS production and the ROS-producing gene *moladietz*, itself a JNK signalling target, and hence a feedback loop was formed (Khan et al., 2017; Pinal et al., 2018). As we have mentioned, ROS production can also occur during CIN-induced tumourigenesis, where it is thought to activate JNK signalling via Ask1 activity (Figure 5; Muzzopappa et al., 2017).

### SWH Signalling

We have discussed how JNK signalling can differentially regulate the SWH signalling pathway – upregulating Yki during compensatory proliferation and tumourigenesis (Doggett et al., 2011; Ohsawa et al., 2011; Sun and Irvine, 2011, 2013; Enomoto et al., 2015), and downregulating Yki during cell competition and tissue growth regulation (Doggett et al., 2011; Chen et al., 2012). However, it has also been recently shown that the opposite is possible – SWH signalling is capable of regulating JNK signalling. Yki/Sd activity was shown to promote tissue overgrowth, as well as *Rho1* transcription which, as we have discussed, is capable of then promoting JNK signalling – specifically, researchers found that Rho1 activated JNK signalling via Tak1 and Hep (Figure 5; Ma et al., 2015a). Interestingly, this interaction was only observed in the wing, and not eye-antennal, imaginal discs, suggesting it is context dependent (Ma et al., 2015a). Furthermore, Yki-mediated overgrowth was rescued by blocking JNK signalling, and was phenocopied via coupling Rho1 and p35 expression (promoting JNK signalling and blocking apoptosis) (Ma et al., 2015a). Conversely, it has also been demonstrated that activating SWH signalling, and hence blocking Yki, can lead to invasive behaviour, which is primarily governed by JNK signalling – indeed, JNK signalling was upregulated and responsible for the invasiveness (Ma et al., 2017). Mechanistically, researchers showed that the Yki target, *bantam* (a microRNA), suppresses *Rox8*, which acts as a positive regulator of JNK signalling, possibly via its role in downregulating *dlg1* (Figure 5; Ma et al., 2017). Therefore, an interesting regulatory situation occurs in the wing epithelium: SWH signalling inactivation promotes tissue growth via Yki-mediated activation of JNK signalling (Ma et al., 2015a), while SWH signalling activation promotes JNK-mediated invasiveness (Ma et al., 2017). Another interesting example of interaction between Yki and JNK signalling occurs during wound healing, a process JNK is well known to be involved in via its capacity as a regulator of cell movement and morphology (Lesch et al., 2010). Researchers found that Yki was required (alongside Sd) for wound closure in larval epidermal tissue not via its canonical roles in proliferation, but rather by regulating actin polymerisation (Tsai et al., 2017). Furthermore, they found that Yki activity during wound closure occurred independently of Pvr signalling, but likely acts parallel to or downstream of JNK signalling (Tsai et al., 2017).

### Other Regulators

Lastly, while these previous examples highlight how JNK signalling can be activated due to certain signalling pathways, or as a result of different biological phenotypes, the pathway can also be induced or repressed by simple genetic lesions. As such, these mutations can potentially contribute significantly to tumourigenesis, and so we feel it is important to call attention to some of the more recently identified examples. JNK signalling can be activated by mutations in genes such as *jumeau* (*jumu*, a Fork head family TF) (Wang et al., 2019) or *pontin* (*pont*, member of the ATPases Associated with various cellular Activities (AAA+) family) (Wang et al., 2018), or by overexpression of genes such as *growth arrest and DNA damage-inducible 45* (*Gadd45*, orthologue of human *GADD45G*) (Camilleri-Robles et al., 2019). Other negative regulators of JNK signalling include the Striatin interacting phosphatase and kinase (STRIPAK) complex members, Connector of kinase to AP-1 (Cka) and Striatin interacting protein (Strip) – when JNK signalling is activated via Immune Deficiency (IMD) signalling, part of the *Drosophila* innate immune system (reviewed in Hoffmann, 2003; Kaneko and Silverman, 2005), these molecules act to suppress JNK pathway activity (Bond and Foley, 2009; Ashton-Beaucage et al., 2014). However, it has also been shown that mutating or knocking down *Cka* or *Strip* during *Drosophila* larval spermatogenesis induces JNK signalling via Egr, independently of IMD signalling, suggesting Cka and Strip may act more universally (La Marca et al., 2019). Conversely, JNK signalling can be inhibited by mutations in genes such as *deltex* (*dx*, a positive regulator of Notch signalling) (Dutta et al., 2018), *Glycine N-acyltransferase* (*Glyat*, orthologue of human GLYATL3) (Ren et al., 2017), and various members of the Toll (a.k.a. NF-κB) signalling pathway (Wu et al., 2015). Interestingly, while Toll signalling is necessary for JNK-mediated cell death (Wu et al., 2015), it is repressed in *scrib*^–/–^ cell competition where, if activated, Toll signalling in *scrib*^–/–^ cells then occurs alongside simultaneous activation of JNK signalling and accumulation of F-actin, which promotes Yki activity and tumourigenesis (Katsukawa et al., 2018). Some more unusual examples are genes that promote JNK signalling when overexpressed, but inhibit it when mutated, such as *licorne* (*lic*, orthologue of human *MAP2K6*) (Sun et al., 2019) or *tankyrase* (*Tnks*, encodes NAD(+) ADP-ribosyltransferase) (Feng et al., 2018).

In summary, a wide variety of activation contexts exist for JNK signalling, each of which seem to drive markedly different cellular behaviours and outcomes. Direct regulation of the JNKKKs appears to be a key method for inducing different roles for the pathway, and occurs through mechanisms as diverse as actin cytoskeleton regulation and ROS production. The Rho-family GTPases are, in particular, granted a key role in these non-canonical activations, and the pathway is also capable of activating itself via a number of different feedback loops. Many more JNK-activating signals are doubtless waiting to be discovered.

## Conclusion and Perspectives

Jun N-terminal kinase signalling is a complex process. An intricate array of upstream signalling molecules feed into the activation of the single titular JNK in *Drosophila*, Bsk, which then activates an equally vast and detailed collection of downstream TFs and target genes. However, this highly conserved pathway is significantly more complex in mammals and, hence, the relative simplicity of JNK signalling in *Drosophila*, coupled with the powerful genetic techniques available to researchers using this model organism, means *Drosophila* has been (and will doubtless continue to be) an indispensable tool in uncovering the molecular basis for the two-faced nature of JNK: being both pro- and anti-tumourigenic. There has been a massive increase in our understanding of these processes over the last decade, which we have attempted to capture in this review. This final section will look at some of the future directions the field may take, as informed by a number of recent and unique explorations of JNK signalling.

While the upstream complexity of JNK signalling contributes to the power of the pathway to drive such varied outcomes, it is also an obstacle to obtaining a comprehensive understanding of its biological contributions. Further complications arise due to the possibility that components of the pathway are effecting roles unrelated to the central JNK signalling cascade. One such example has been shown for Egr – in tumourigenic tissue wholly mutant for *dlg1*, Egr secreted by attracted haemocytes sensitises the cells of the tumour to activity of the antimicrobial peptide Defensin, which promotes tumour cell death (Parvy et al., 2019). Though a role for JNK signalling in the process was not explicitly ruled out, the possibility of more JNK-unrelated roles for JNK pathway members lends itself to potential new avenues of research.

Alongside the various upstream actuators of JNK signalling, there is considerable potential to explore how the intensity of the JNK signal affects its pro- and anti-tumourigenic properties. As discussed, one recent study found that Cno overexpression led to JNK-mediated tissue overgrowth, but slightly modulating JNK signalling then led to massive overgrowth (Ma et al., 2019). The researchers determined that Cno overexpression upregulated both JNK and Ras-MAPK signalling, each of which downregulated SWH signalling to promote Yki-dependent tissue growth, and suggested that subsequent modulation of JNK signalling inhibited its anti-tumourigenic role in promoting apoptosis, while leaving its pro-tumourigenic role as a SWH pathway inhibitor intact (Ma et al., 2019). In a similar vein another recent study examined JNK signalling levels in response to tissue damage. It was shown that high levels of ROS produced in damaged tissue phosphorylated and activated Ask1 and, therefore, strong JNK signalling and apoptosis (Santabárbara-Ruiz et al., 2019). However, it was found that the ROS signal propagated to undamaged neighbouring tissue, and Ask1 was then phosphorylated by both the ROS signal and Akt1, a downstream protein kinase of the PI3K signalling pathway (Santabárbara-Ruiz et al., 2019). This altered activation context for Ask1 led to a lower level of JNK signal, which promoted cell proliferation and survival rather than apoptosis (Santabárbara-Ruiz et al., 2019). Both these studies are examples of the potential for altering JNK signalling levels to profoundly alter the outcome of the pathway, and are exemplars of an exciting new avenue of research regarding pro- and anti-tumourigenic JNK signalling.

The importance of subcellular localisation of JNK signalling components is also an area that is ripe for exploration. A critical role for endocytosis in JNK signalling has been demonstrated, with increased endocytosis thought to be key in upregulating the TNF-JNK signalling observed in *scrib*^–/–^ clones (Igaki et al., 2009). Furthermore, another study has shown Rho1 specifically localised to the cell membrane can activate apoptosis-inducing JNK signalling that acts primarily via Slpr and Tak1 and, indeed, regulates the subcellular localisation of Slpr via physical interaction (Neisch et al., 2010).

The relationship between JNK and SWH signalling is, as yet, unresolved. There is clearly complex interplay between the pathways, highly dependent on cellular context, but its elucidation is critical for obtaining a more complete understanding of pro- and anti-tumourigenic JNK signalling. While recent research discussed in this review has undoubtedly advanced our knowledge greatly (Doggett et al., 2011; Sun and Irvine, 2011; Chen et al., 2012; Enomoto et al., 2015; Ma et al., 2015a, 2017; Gerlach et al., 2018), there is still much to be discovered, particularly with regard to how the pathways interact during cooperative tumourigenesis. This relationship between JNK and SWH signalling is undoubtedly one that will be examined closely in coming years.

There is a preponderance of different model systems used in the study of JNK signalling within *Drosophila* (e.g., different tissues and tissue regions, different mutant clones, different models of cooperative tumourigenesis). While this is certainly a great strength of the organism as a model, it is also a complicating factor, making it difficult to generate a cogent, unified model for JNK signalling (if this indeed exists). While the exploration of JNK signalling in a wide variety of contexts is undoubtedly beneficial, and should be encouraged and highlighted, we believe that a discussion regarding standardised systems in which observations regarding JNK signalling might be examined and replicated is worthwhile.

Finally, it is worth reiterating the relevance of *Drosophila* studies to explorations of mammalian genetics and disease. We have touched upon how JNK signalling has two faces in humans, as it does in flies – it can be both pro- and anti-tumourigenic (or pro- and anti-survival), depending on the context (reviewed in Wagner and Nebreda, 2009; Wu et al., 2019). The inherently increased complexity of human JNK signalling makes the pathway difficult to work with, however, and this leaves *Drosophila* research at an advantage, able to use parallels between the highly conserved systems to position itself as a foundational body of knowledge for the field – conclusions drawn in flies can be adapted and extended into mammalian research. For example, we have discussed how Rho-family GTPases have emerged as key activators of JNK signalling in *Drosophila* (Garlena et al., 2010; Brumby et al., 2011; Khoo et al., 2013; Rudrapatna et al., 2014; Ma et al., 2016), and similar conclusions have been reached in mammals – *RAC1* gain-of-function mutations are common in *BRAF* and *NRAS*-driven melanomas, and said mutations can drive RAC1 binding to and activation of targets like PAK1 (p21 (RAC1) activated kinase 1) and MAP3K11 (mitogen-activated protein kinase kinase kinase 11, a.k.a. MLK3) (Krauthammer et al., 2012). Both PAK1 (Qing et al., 2012) and MAP3K11 (reviewed in Gallo and Johnson, 2002) have been shown to be activators of JNK signalling in mammals. Interestingly, while the *Drosophila* PAK1 orthologue, p21-activated kinase (Pak), is not known to act via JNK signalling (Conder et al., 2004), the orthologue of MAP3K11 is Slpr, thought to be the key JNKKK in cell morphological changes and embryogenesis processes like dorsal closure (reviewed in Ríos-Barrera and Riesgo-Escovar, 2013). Another study examining *BRAF*-driven melanoma found that ectopic JNK signalling via JUN was likely partly responsible for the invasiveness of the tumour cells (Ramsdale et al., 2015). As discussed, JUN is one half of the AP-1 TF complex, alongside FOS, which is conserved in *Drosophila*. Furthermore, pharmacologically inhibiting both BRAF and JNK signalling proved an effective way of suppressing invasiveness and increasing tumour cell death (Ramsdale et al., 2015). This example is clearly reminiscent of the invasive tumourigenesis seen when overactive Ras-MAPK and JNK signalling are coupled in *Drosophila*, as discussed throughout this review.

While a direct connection has not yet been made between JNK signalling research in *Drosophila* and the development of human cancer therapeutics, the JNK signalling pathway is clearly two-faced during tumourigenesis in both flies and humans (Supplementary Table 2), and therefore the extensive research generated in the fly model may serve to inform which types of human cancer types are likely to be JNK-dependent, in order to triage these patients for treatment with JNK pathway inhibitors. For example, genetic analyses of the *Drosophila* Ret oncogene (Ret) protein tyrosine kinase pathway revealed that the JNK pathway was anti-tumourigenic, which was paralleled in cases of the human Ret-driven cancer, multiple endocrine neoplasia type 2 (Read et al., 2005), thereby implying that targeting the JNK pathway in these cancers would not be beneficial. There has been considerable interest in developing drugs that target JNK signalling for use in the treatment of cancers (and other diseases), and some success has been obtained, but the pathway is so context dependent, with such a diverse array of targets and effectors, that determining how best to exploit the pathway pharmacologically can be difficult (reviewed in Messoussi et al., 2014; Cicenas, 2015; Cicenas et al., 2017). Nonetheless, research into *Drosophila* JNK signalling has, does, and will continue to support our understanding of JNK signalling in humans but, as it relates to cancer progression, more research is clearly needed. With the advent of transgenic RNAi (reviewed in Perrimon et al., 2010) and, more recently, CRISPR/Cas9 technology in *Drosophila* (reviewed in Housden and Perrimon, 2016; Bier et al., 2018), the generation of more sophisticated models of human cancer in flies has been possible, which can be used for chemical screening for anti-cancer compounds (Richardson et al., 2015; Sonoshita and Cagan, 2017; Cagan et al., 2019), a process already achieving promising results (Bangi et al., 2016, 2019). Similar methodologies using *Drosophila* as avatars for specific human cancers could be applied specifically to investigate whether the JNK pathway is pro- or anti-tumourigenic in particular contexts, and how it might be targeted to be beneficial therapeutically. Moreover, *Drosophila* JNK-dependent cancer models can be used in the development of JNK pathway inhibitors that are more bioavailable, and which have greater efficacy and lower toxicity, as has been achieved using a *Drosophila* model of Ret-driven cancer (Dar et al., 2012; Sonoshita et al., 2018; Ung et al., 2019). With the adoption of these new technologies, the *Drosophila* model still has much to offer toward the understanding and targeting of the JNK pathway in human cancers.

## Author Contributions

JL wrote the manuscript and prepared the figures. HR provided intellectual direction and editorial advice.

## Conflict of Interest

The authors declare that the research was conducted in the absence of any commercial or financial relationships that could be construed as a potential conflict of interest.
