# Supplementary material for: Two-Faced: Roles of JNK Signalling During Tumourigenesis in the Drosophila Model
Source: Front Cell Dev Biol. 2020 Feb 5;8:42. doi: 10.3389/fcell.2020.00042 (PMC7012784; doi:10.3389/fcell.2020.00042)
Supplement: Supplementary file 1 [file Table_1.docx]

Supplementary Material

**Supplementary Table 1. Abbreviations and glossary.** A full list of abbreviations used multiple times outside their original context in this review, and a glossary of those terms.

| **Abbreviation** | **Full Name** | **Notes** |
| --- | --- | --- |
| AP-1 | Activator Protein-1 | Name of complex formed by Jra and Kay in *Drosophila* and their orthologues in mammals |
| aPKC | atypical Protein Kinase C | *Drosophila* apico-basal polarity regulator protein, human orthologues in PRKCI and PCKCZ |
| Ask1 | Apoptotic signal-regulating kinase 1 | *Drosophila* JNKKK, human orthologues in MAP3K15 and MAP3K5 |
| Bsk | Basket | *Drosophila* JNK, human orthologues in MAPK10, MAPK8, and MAPK9 |
| Cher | Cheerio | *Drosophila* F-actin crosslinking protein, human orthologues in FLNA/B/C |
| CIN | chromosomal instability | Phenomenon whereby whole chromosomes or sections thereof duplicate, leading to aneuploidy and potentially tumourigenesis |
| Cno | Canoe | *Drosophila* adherens junction scaffold protein, human orthologue AFDN |
| CycE | Cyclin E | *Drosophila* cell cycle regulator protein, human orthologues in CCNE1/2 |
| Diap1 | Death-associated inhibitor of apoptosis 1 | *Drosophila* apoptosis-inhibiting protein |
| Dlg1 | Discs large 1 | *Drosophila* apico-basal polarity regulator protein, human orthologues in DLG1, DLG2, DLG4, and DLG3 |
| Dpp | Decapentaplegic | *Drosophila* TGF-β ligand, human orthologues in BMP2 and BMP4 |
| Egr | Eiger | *Drosophila* TNF pathway ligand |
| Ena | Enabled | *Drosophila* actin polymerase, human orthologues in ENAH, EVL, and VASP |
| FOS | Fos proto-oncogene, AP-1 transcription factor subunit | Human orthologue of Kay |
| Grnd | Grindelwald | TNF signalling pathway receptor, activated by Egr |
| Hep | Hemipterous | *Drosophila* JNKK, human orthologues in MAP2K4 |
| Hid | Head involution defective | *Drosophila* apoptosis-promoting protein |
| Jak-STAT | Janus kinase-Signal Transduction and Activator of Transcription | Signalling pathway with a diversity of roles, but often involved in intercellular signalling |
| JNK | c-Jun N-terminal Kinase | JNK pathway kinase, activated by JNKKs, and that induces TF activity, also a signalling pathway commonly involved in apoptosis and proliferation regulation |
| JNKK | c-Jun N-terminal Kinase Kinase | JNK pathway kinase kinase, activated by JNKKKs, and that activate JNKs |
| JNKKK | c-Jun N-terminal Kinase Kinase Kinase | JNK pathway kinase kinase kinase, activated by JNKKKKs, and that activate JNKKs |
| Jra | Jun-related antigen | TF downstream of JNK signalling in *Drosophila*, forms AP-1 complex with Kay, human orthologues in JUN and JUND |
| Jub | Ajuba LIM protein | *Drosophila* Wts-inhibiting protein, human orthologues in LIMD1, WTIP, and AJUBA |
| JUN | Jun proto-oncogene, AP-1 transcription factor subunit | Human orthologue of Jra |
| Kay | Kayak | TF downstream of JNK signalling in *Drosophila*, forms AP-1 complex with Jra, human orthologues in FOS, FOSL1, and FOSL2 |
| L(2)gl | Lethal (2) giant larvae | *Drosophila* apico-basal polarity regulator protein, human orthologues in LLGL1 and LLGL2 |
| MAPK | Mitogen Activated Protein Kinase | Class of protein kinase of which JNK is a member, also commonly used as a moniker for signalling pathways proceeding via Egfr and/or Ras85D in *Drosophila* |
| Mbc | Myoblast city | *Drosophila* guanine nucleotide exchange factor protein |
| Mkk4 | MAP kinase kinase 4 | *Drosophila* JNKK, human orthologues in MAP2K7 |
| Mmp1 | Matrix metalloproteinase 1 | *Drosophila* extracellular proteinase, common target of JNK signalling, closest human orthologues in MMP14/24 |
| nTSGs | neoplastic tumour suppressor genes | Class of genes where their inactivation leads to neoplastic tumour growth, characterized by tissue overproliferation and aberrant differentiation |
| PI3K | Phosphoinositide 3-kinase | Signalling pathway, also known as the AKT or mTOR pathway, with a diversity of roles, but often involved in cell cycle regulation |
| Pvr | PDGF- and VEGF-receptor related | *Drosophila* receptor protein, capable of activating a range of signalling pathways, including PI3K and JNK |
| Raf | Raf oncogene | *Drosophila* kinase protein, lying downstream of Ras85D, human orthologues in BRAF, ARAF, and RAF1 |
| Ras85D | Ras oncogene at 85D | *Drosophila* GTPase protein, human orthologues in HRAS, KRAS, and NRAS |
| Robo2 | Roundabout 2 | *Drosophila* receptor protein, human orthologues in ROBO1/2/3/4 |
| ROS | reactive oxygen species | Reactive chemical species containing oxygen which can be damaging biologically, and can be produced due to cellular stress |
| Rpr | Reaper | *Drosophila* apoptosis-promoting protein |
| Scrib | Scribble | *Drosophila* apico-basal polarity regulator protein, human orthologue SCRIB |
| Sd | Scalloped | *Drosophila* TF active alongside Yki, human orthologues in TEAD1/3/4/2 |
| Shg | Shotgun | *Drosophila* protein also known as E-cadherin |
| Slpr | Slipper | *Drosophila* JNKKK, human orthologues in MAP3K9, MAP3K10, MAP3K11, and MAP3K21 |
| Src42A | Src oncogene at 42A | *Drosophila* kinase protein, human orthologue FRK |
| Src64B | Src oncogene at 64B | *Drosophila* kinase protein, human orthologues in FYN and SRC |
| SWH | Salvador-Warts-Hippo | Signalling pathway also referred to as the Hippo pathway, suppresses tissue growth by downregulating Yki activity |
| Syx7 | Syntaxin 7 | *Drosophila* endocytic process protein, human orthologues in STX7/12 |
| Tak1 | TGFβ-associated kinase 1 | *Drosophila* JNKKK, human orthologue MAP3K7 |
| TFs | transcription factors | Class of proteins that contribute to gene transcription |
| TGF-β | Transforming Growth Factor-β | Signalling pathway with a diversity of roles, but often involved in cell growth and proliferation |
| TNF | Tumour Necrosis Factor | Signalling pathway capable of activating JNK signalling in *Drosophila* |
| Upd1/2/3 | Unpaired 1/2/3 | *Drosophila* Jak-STAT signalling pathway ligands |
| Wg | Wingless | Signalling pathway also known as the WNT pathway with a diversity of roles in *Drosophila*, but commonly involved in cellular communication and embryogenesis |
| Wgn | Wengen | TNF signalling pathway receptor, activated by Egr |
| Wnd | Wallenda | *Drosophila* JNKKK, human orthologues in MAP3K13 and MAP3K12 |
| Wts | Warts | *Drosophila* kinase protein from the SWH pathway, human orthologues in LATS1/2 |
| Yki | Yorkie | *Drosophila* transcriptional co-activator downregulated by SWH signalling, human orthologue in YAP1 |

**Supplementary Table 2. Conservation of the JNK pathway between *Drosophila melanogaster* and *Homo sapiens*.** The core JNK signalling pathway components in *Drosophila* have orthologues in humans, but with greater genetic redundancy, as is often the case. Note also there are many links between human JNK components and tumourigenesis – here we provide an overview of the various cancers and cancer models to which the human orthologues have been directly linked. However, such links are certain to only scratch the surface regarding how the versatile JNK signalling pathway can affect cancer development in more fundamental ways. We have included the most recent studies for each the human genes where possible, but the field is vast, and we are unable to cover it in its entirety, and so note that it can also be found reviewed in Wagner and Nebreda (2009), Kyriakis and Avruch (2012), Kitanaka et al. (2013), Tournier (2013), Bubici and Papa (2014), Li et al. (2016), Wang and Tai (2016), Yan et al. (2016), Dhanasekaran and Reddy (2017), Wu et al. (2019), Xu and Hu (2020), and Gallo et al. (in press). Note that many more studies than we have cited here link JNK signalling to cancer, but some only explore JNK in a general way, and we tried to limit the studies we have cited to those that were explicit in which JNK pathway gene(s) they examined.

| **JNK Pathway Role** | ***Drosophila* Genes** | **Human Orthologues** | **Known Links to Human Tumourigenesis** |
| --- | --- | --- | --- |
| JNKKKs | *slpr* | *MAP3K9* | - Inactivating mutations found in 24% of tested melanoma cell lines (alongside *MAP3K5*) (Stark et al., 2011) - Activating mutations found in some non-small cell lung cancers (Fawdar et al., 2013) - Overexpressed in renal carcinomas, repressed by MicroRNA-148b (Nie et al., 2016) |
|  |  | *MAP3K10* | - Upregulation promotes proliferation of pancreatic ductal adenocarcinoma cells, and inhibits their sensitivity to gemcitabine treatment (An et al., 2013) - Likely downregulated by MicroRNA-155 activity in osteosarcoma (Wang et al., 2017a) |
|  |  | *MAP3K11* | - Dominant-negative MAP3K11 expression suppresses hepatoma cell death (Kim et al., 2004) - Required generally for growth and invasion of various cancer cells (Chen et al., 2010; Chen and Gallo, 2012; Whitworth et al., 2012; Zhan et al., 2012) - The oncogenic P252H mutation of MAP3K11 is common in gastrointestinal cancers, and upregulates Wnt, Notch, and MAPK signalling pathways (Velho et al., 2010; Corso et al., 2011; Velho et al., 2014) - Ectopically activated in Human epidermal growth factor 2 (HER2)-positive cancers, but pro-apoptotic functionality is suppressed by HER2 and restored via drug treatment (Das et al., 2015) - MicroRNA-199a95p expression suppresses oesophageal tumourigenesis by repressing *MAP3K11* (Byrnes et al., 2016) - Glioblastoma invasiveness driven by EGFR signalling via DOCK1 and MAP3K11 (Misek et al., 2017) - Activated in a positive feedback loop with ERK1/2 via oxidative stress to promote colorectal tumourigenesis and invasion (Schroyer et al., 2018) - Promotes breast tumourigenesis via phosphorylation of p21-activated kinase 1 (Das et al., 2019) |
|  |  | *MAP3K21* | - Commonly mutated in in colorectal cancers, where constitutively active alleles can promote tumourigenesis alongside activated KRAS or BRAF (Martini et al., 2013) - Loss-of-function mutations in colorectal cancer cells reduce growth rates and tumour sizes (Marusiak et al., 2016) - Found to be overexpressed in triple-negative breast cancers, where it promotes invasiveness (Marusiak et al., 2019) |
|  | *wnd* | *MAP3K13* | - Inactivating mutations in *MAP3K13* have been identified as somatic driver mutations promoting oncogenesis in breast cancers (Stephens et al., 2012) |
|  |  | *MAP3K12* |  |
|  | *Tak1* | *MAP3K7* | - Breast tumourigenesis and metastasis can proceed via MAP3K7 activity stimulated by TGF-β signalling (Neil and Schiemann, 2008; Safina et al., 2008) - Metastatic breast cancers often invade bones, a process mediated by MAP3K7 signalling activity (Safina et al., 2011) - Necessary in some colon cancers with activated RAS signalling (Singh et al., 2012) - EMT of skin squamous cell carcinoma is promoted by MAP3K7 downregulation, which is common in said cancers (Lam et al., 2013) - Overexpression correlates with metastatic tumour behaviour and poor survival in oesophageal squamous cell carcinomas and clear cell renal cell carcinomas (Wei et al., 2013; Wen et al., 2013) - Overexpressed in renal cell carcinomas, where it acts as an oncogene, upregulating NF-κB signalling (Fandong et al., 2014) - Overexpressed in many thyroid cancers, with the level of said overexpression correlating with cancer metastasis (Lin et al., 2015) - Deletion/suppression of *MAP3K7* is strongly associated with prostate tumourigenesis, and also synergises with CHD1 suppression to lead to enhanced cooperative prostate tumourigenesis (Wu et al., 2012; Kluth et al., 2013; Rodrigues et al., 2015) - Necessary for the survival of lung cancer cells, where it is activated downstream of TGF-β/BMP signalling (Augeri et al., 2016) - Lies downstream of various metastasis-promoting genes, such as *UBE2N* in breast cancer and *MET* in hepatocellular carcinoma (Wu et al., 2014; Tey et al., 2017) - Overexpressed in gastric cancer, and its level correlates with reduced survival rates and disease progression (Yang et al., 2017) - Tumour suppressor role of MicroRNA-146a in gastric cancer due to its targeted inhibition of *MAP3K7* (Chen et al., 2017) - Upregulation in pancreatic ductal adenocarcinoma associated with apoptosis suppression and lymph node metastasis (Melisi et al., 2011; Huang et al., 2017a) - Overexpressed in many breast cancers, and inhibition of MAP3K7 suppresses triple-negative breast cancer metastasis to the lungs in animal models (Huang et al., 2015; Iriondo et al., 2018) - Commonly constitutively active in cutaneous T-cell lymphoma cells, and acts upstream of NF-κB and β-catenin signalling (Gallardo et al., 2018) |
|  | *Ask1* | *MAP3K15* |  |
|  |  | *MAP3K5* | - Found to be commonly mutated in melanoma samples, where the R256C mutation led to increased proliferation, and apoptosis is induced by MAP3K5 in response to novel atmospheric gas plasma treatment (Ishaq et al., 2014; Prickett et al., 2014) - CLDN6 acts as a breast cancer tumour suppressor, and this effect correlates with MAP3K5 expression in multiple cancers (Guo et al., 2012; Zhang et al., 2015; Guo et al., 2016) - Lung adenocarcinoma cells survive based on downregulation of MAP3K5 activity by ROR1 (Ida et al., 2016) - Multiple drugs activate MAP3K5 signalling in hepatocellular carcinoma cells, where it is known to promote apoptosis – 4SC-202 does so independently, while ABT-737 synergises with curcumin (Fu et al., 2016; Jiang et al., 2016; Zhang et al., 2016; Zheng et al., 2016) - Deletion in platelets non-autonomously reduces lung cancer metastasis (Kamiyama et al., 2017) - In neuroblastoma cells, MAP3K5 is active and stabilises NR2E1, which promotes cell survival (Sobhan et al., 2017) - Sensitivity of colorectal cancer cells to drug treatment was enhanced after MAP3K5 was derepressed (Zhang et al., 2018) - SLC35F2 is commonly overexpressed in cancers, and in papillary thyroid carcinoma promotes tumourigenesis via MAP3K5 (He et al., 2018) - Overexpression in gastric cancer cells inhibits proliferative and migratory behaviours via JNK and p38 signalling (Wan et al., 2018) |
| JNKKs | *hep* | *MAP2K4* | - Implicated as having tumour suppressor functionality in both ovarian cancer and lung adenocarcinoma (Ahn et al., 2011; Yeasmin et al., 2011) - Expression strongly correlates with multiple osteosarcoma progression parameters, as well as cell proliferation in pancreatic ductal adenocarcinoma (Handra-Luca et al., 2012; Tesser-Gamba et al., 2012) - Promotes metastatic behaviour independently of JNK or p38 downstream targets in some prostate cancers (Pavese et al., 2014) - MicroRNA-27a downregulates *MAP2K4* – in osteosarcoma cell lines this then promotes tumourigenic behaviour, but in prostate cell lines this suppresses it (Pan et al., 2014; Wan et al., 2016) - Low MAP2K4 activity, as measured by phosphorylation, is correlated with increases metastatic capability in colorectal cancer samples (Wang et al., 2017b) - MicroRNA-802 targets *MAP2K4*, but is downregulated in some tongue squamous cell carcinomas, contributing to tumourigenesis (Wu et al., 2017) - Resistance of triple-negative breast cancers to PI3K signal inhibition has been linked to MAP2K4 activity (Mundt et al., 2018) - High MAP2K4 (alongside MAP2K7) expression is linked to favourable prognoses in some pancreatic ductal adenocarcinomas (Lu et al., 2019) |
|  | *Mkk4* | *MAP2K7* | - Hepatoma treatment via flavonoid drugs occurs in part via the induction of MAP2K7 activity (Tang et al., 2012) - Elevated MAP2K7 activity contributes to hepatocellular carcinoma tumourigenesis (Guo et al., 2013) - MicroRNA-493-based inhibition of *MAP2K7* likely contributes to preventing the metastasis of colon cancers to the liver (Sakai et al., 2014) - Drug-based suppression of the capacity of multiple myeloma to suppress MAP2K7-mediated apoptosis may be a promising treatment avenue (Tornatore et al., 2014) - MAP2K7 is thought to play a role in breast cancer development, where the post-translational modification of neddylation regulates its activity (Zhu et al., 2016) - Certain *MAP2K7* alleles may indicate a higher risk of certain lung cancers (Qiu et al., 2016; Jia et al., 2017) - High MAP2K7 (alongside MAP2K4) expression is linked to favourable prognoses in some pancreatic ductal adenocarcinomas (Lu et al., 2019) - A review of the potential of MAP2K7 as a therapeutic target in cancer has also recently been published (Park et al., 2019) |
| JNKs | *bsk* | *MAPK10* | - Tumour suppressor CDKN2A acts to inhibit MAPK10 activity and so prevents tumourigenesis (Choi et al., 2005) - May act as a tumour suppressor in chromophobe renal cell carcinoma depending on its epigenetic status (Yoo et al., 2011) - Expression positively correlates with disease progression in various cancers (Dai et al., 2014) - MicroRNA-27a-3p downregulates *MAPK10* to promote tumourigenesis in nasopharyngeal carcinoma (Li and Luo, 2017) |
|  |  | *MAPK8* | - Estrogen signalling may act alongside JNK signalling via MAPK8 activity to affect breast cancer and cervical epithelial cancer progression (Fogarty et al., 2012; Sun et al., 2012) - Multidrug resistance of colon cancer is mediated in part by JNK signalling via MAPK8 (Zhu et al., 2012) - Stromal MAPK8 signalling in breast cancers is highly important in their progression and in developing malignance (Lisanti et al., 2014) - Pro-apoptotic MAPK8 signalling is suppressed in hepatocellular carcinoma to facilitate aerobic glycolysis (Iansante et al., 2015) - FXR suppresses MAPK8 activity to inhibit chemically-induced liver tumourigenesis (Wang et al., 2015) - MAPK8 expression positively regulates vitamin D receptor expression, which then facilitates vitamin D-mediated colorectal cancer cell proliferation inhibition (Bi et al., 2016) - JNK signalling via MAPK8 (and MAPK9) is activated by hepatitis B virus X protein, and overcomes tumour suppressor TGF-β signalling in hepatocellular carcinoma (Wu et al., 2016) - Gastric cancer cells undergo apoptosis after dihydroartemisinin administration via JNK signalling through MAPK8 (and MAPK9) (Zhang et al., 2017) - JNK signalling through MAPK8 (and MAPK9) activates Notch signalling in triple-negative breast cancer cells to promote tumourigenesis (Xie et al., 2017) - Expression is reduced in oesophageal squamous cell carcinomas, and expression in the stromal cells specifically correlates with tumourigenesis (Bao et al., 2018) |
|  |  | *MAPK9* | - Overexpressed in many non-small cell lung carcinomas, and is necessary for their growth, as well as in some cases their capacity to initiate tumourigenesis (Nitta et al., 2011; Okada et al., 2013) - Decreasing MAPK9 activity may contribute to the effectiveness of ulinastatin on inhibiting breast cancer cell growth (Wang et al., 2012) - Constitutively activated MAPK9 is found and necessary in various multiple myeloma tumours (Barbarulo et al., 2013) - MAPK9 (and MAPK8) may be necessary for the resistance of prostate cancers to certain drug treatments (Parra and Ferreira, 2013) - JNK signalling via MAPK9 and downstream of KRAS contribute to tumour-initiation by pancreatic cancer stem cells (Okada et al., 2014) - Colorectal cancer metastasis may depend in part on downregulation of MicroRNA-200c and the concomitant upregulation of *MAPK9* (Sui et al., 2014) - MAPK9 (and MAPK8) contribute to the capacity of chemoresistant cell lines to initiate tumourigenesis (Liu et al., 2015) - Non-small cell lung carcinonoma cells undergo apoptosis after MAPK9 (and MAPK8) upregulation in response to phloretin treatment (Min et al., 2015) - MAPK9 is misregulated in ulcerative colitis, which can lead to cancer, but restoring its functionality might be preventative with regards to this tumourigenesis (Lessel et al., 2017; Reissig et al., 2017) - Upregulating *MAPK9* by downregulating MicroRNA-200c can contribute to bladder cancer invasiveness (Huang et al., 2017b) - MAPK9 (and MAPK8) contribute to osteosarcoma invasiveness, which is suppressed by inhibiting their activity (Lu et al., 2018) - MAPK9 (and MAPK8) activity is suppressed in many lung squamous cell carcinomas, with increased activity being indicative of better survival rates amongst similar cancers (Liu et al., 2019) - Contributes to melanoma cell tumourigenesis and drug resistance (Du et al., 2019) |

**References**

Ahn, Y.H., Yang, Y., Gibbons, D.L., Creighton, C.J., Yang, F., Wistuba, I.I., et al. (2011). *Map2k4* functions as a tumor suppressor in lung adenocarcinoma and inhibits tumor cell invasion by decreasing peroxisome proliferator-activated receptor γ2 expression. *Molecular and Cellular Biology* 31(21)**,** 4270-4285. doi: <https://doi.org/10.1128/mcb.05562-11>.

An, Y., Cai, B., Chen, J., Lv, N., Yao, J., Xue, X., et al. (2013). MAP3K10 promotes the proliferation and decreases the sensitivity of pancreatic cancer cells to gemcitabine by upregulating Gli-1 and Gli-2. *Cancer Letters* 329(2)**,** 228-235. doi: <https://doi.org/10.1016/j.canlet.2012.11.005>.

Augeri, D.J., Langenfeld, E., Castle, M., Gilleran, J.A., and Langenfeld, J. (2016). Inhibition of BMP and of TGFβ receptors downregulates expression of XIAP and TAK1 leading to lung cancer cell death. *Molecular Cancer* 15. doi: <https://doi.org/10.1186/s12943-016-0511-9>.

Bao, Y., Zhang, S., Guo, Y., Wei, X., Zhang, Y., Yang, Y., et al. (2018). Stromal expression of JNK1 and VDR is associated with the prognosis of esophageal squamous cell carcinoma. *Clinical and Translational Oncology* 20**,** 1185-1195. doi: <https://doi.org/10.1007/s12094-018-1843-2>.

Barbarulo, A., Iansante, V., Chaidos, A., Naresh, K., Rahemtulla, A., Franzoso, G., et al. (2013). Poly(ADP-ribose) polymerase family member 14 (PARP14) is a novel effector of the JNK2-dependent pro-survival signal in multiple myeloma. *Oncogene* 32**,** 4231-4242. doi: <https://doi.org/10.1038/onc.2012.448>.

Bi, X., Shi, Q., Zhang, H., Bao, Y., Hu, D., Pohl, N., et al. (2016). c-Jun NH2-teminal kinase 1 interacts with vitamin D receptor and affects vitamin D-mediated inhibition of cancer cell proliferation. *Journal of Steroid Biochemistry and Molecular Biology* 163**,** 164-172. doi: <https://doi.org/10.1016/j.jsbmb.2016.05.009>.

Bubici, C., and Papa, S. (2014). JNK signalling in cancer: in need of new, smarter therapeutic targets. *British Journal of Pharmacology* 171(1)**,** 24-37. doi: <https://doi.org/10.1111/bph.12432>.

Byrnes, K.A., Phatak, P., Mansour, D., Xiao, L., Zou, T., Rao, J.N., et al. (2016). Overexpression of miR-199a-5p decreases esophageal cancer cell proliferation through repression of mitogen-activated protein kinase kinase kinase-11 (MAP3K11). *Oncotarget* 7(8)**,** 8756-8770. doi: <https://doi.org/10.18632/oncotarget.6752>.

Chen, J., and Gallo, K.A. (2012). MLK3 Regulates Paxillin Phosphorylation in Chemokine-Mediated Breast Cancer Cell Migration and Invasion to Drive Metastasis. *Molecular and Cellular Pathobiology* 72(16)**,** 4130-4140. doi: <https://doi.org/10.1158/0008-5472.CAN-12-0655>.

Chen, J., Miller, E.M., and Gallo, K.A. (2010). MLK3 is critical for breast cancer cell migration and promotes a malignant phenotype in mammary epithelial cells. *Oncogene* 29**,** 4399-4411. doi: <https://doi.org/10.1038/onc.2010.198>.

Chen, Y., Zhou, B., Xu, L., Fan, H., Xie, J., and Wang, D. (2017). MicroRNA-146a promotes gastric cancer cell apoptosis by targeting transforming growth factor β-activated kinase 1. *Molecular Medicine Reports* 16**,** 755-763. doi: <https://doi.org/10.3892/mmr.2017.6640>.

Choi, B.Y., Choi, H.S., Ko, K., Cho, Y.-Y., Zhu, F., Kang, B.S., et al. (2005). The tumor suppressor p16^INK4a^ prevents cell transformation through inhibition of c-Jun phosphorylation and AP-1 activity. *Nature Structural & Molecular Biology* 12**,** 699-707. doi: <https://doi.org/10.1038/nsmb960>.

Corso, G., Velho, S., Paredes, J., Pedrazzani, C., Martins, D., Milanezi, F., et al. (2011). Oncogenic mutations in gastric cancer with microsatellite instability. *European Journal of Cancer* 47(3)**,** 443-451. doi: <https://doi.org/10.1016/j.ejca.2010.09.008>.

Dai, F., Zhang, Y., and Chen, Y. (2014). Involvement of miR-29b signaling in the sensitivity to chemotherapy in patients with ovarian carcinoma. *Human Pathology* 45(6)**,** 1285-1293. doi: <https://doi.org/10.1016/j.humpath.2014.02.008>.

Das, S., Nair, R.S., Mishra, R., Sondarva, G., Viswakarma, N., Abdelkarim, H., et al. (2019). Mixed lineage kinase 3 promotes breast tumorigenesis via phosphorylation and activation of p21-activated kinase 1. *Oncogene* 38**,** 3569-3584. doi: <https://doi.org/10.1038/s41388-019-0690-0>.

Das, S., Sondarva, G., Viswakarma, N., Nair, R.S., Osipo, C., Tzivion, G., et al. (2015). Human Epidermal Growth Factor Receptor 2 (HER2) Impedes MLK3 Kinase Activity to Support Breast Cancer Cell Survival. *The Journal of Biological Chemistry* 290(35)**,** 21705-21712. doi: <https://doi.org/10.1074/jbc.M115.655563>.

Dhanasekaran, D.N., and Reddy, E.P. (2017). JNK-signaling: A multiplexing hub in programmed cell death. *Genes & Cancer* 8**,** 682-694. doi: <https://doi.org/10.18632/genesandcancer.155>.

Du, L., Anderson, A., Nguyen, K., Ojeda, S.S., Ortiz-Rivera, I., Nguyen, T.N., et al. (2019). JNK2 Is Required for the Tumorigenic Properties of Melanoma Cells. *ACS Chemical Biology* 14**,** 1426-1435. doi: <https://doi.org/10.1021/acschembio.9b00083>.

Fandong, M., Yan, L., Xin, T., Liye, F., Yuanin, Y., Chengguang, S., et al. (2014). Identification of TGF-β-activated kinase 1 as a possible novel target for renal cell carcinoma intervention. *Biochemical and Biophysical Research Communications* 453**,** 106-111. doi: <https://doi.org/10.1016/j.bbrc.2014.09.070>.

Fawdar, S., Trotter, E.W., Li, Y., Stephenson, N.L., Hanke, F., Marusiak, A.A., et al. (2013). Targeted genetic dependency screen facilitates identification of actionable mutations in FGFR4, MAP3K9, and PAK5 in lung cancer. *Proceedings of the National Academy of Sciences of the United States of America* 110(30)**,** 12426-12431. doi: <https://doi.org/10.1073/pnas.1305207110>.

Fogarty, E.A., Matulis, C.K., and Kraus, W.L. (2012). Activation of estrogen receptor α by raloxifene through an activating protein-1-dependent tethering mechanism in human cervical epithelial cancer cells: A role for c-Jun N-terminal kinase. *Molecular and Cellular Endocrinology* 348**,** 331-338. doi: <https://doi.org/10.1016/j.mce.2011.09.032>.

Fu, M., Wan, F., Li, Z., and Zhang, F. (2016). 4SC-202 activates ASK1-dependent mitochondrial apoptosis pathway to inhibit hepatocellular carcinoma cells. *Biochemical and Biophysical Research Communications* 471(2)**,** 267-273. doi: <https://doi.org/10.1016/j.bbrc.2016.01.030>.

Gallardo, F., Bertran, J., López-Arribillaga, E., González, J., Menéndez, S., Sánchez, I., et al. (2018). Novel phosphorylated TAK1 species with functional impact on NF-κB and β-catenin signaling in human Cutaneous T-cell lymphoma. *Leukemia* 32**,** 2211-2223. doi: <https://doi.org/10.1038/s41375-018-0066-4>.

Gallo, K.A., Ellsworth, E., Stoub, H., and Conrad, S.E. (in press). Therapeutic potential of targeting mixed lineage kinases in cancer and inflammation. *Pharmacology & Therapeutics*. doi: <https://doi.org/10.1016/j.pharmthera.2019.107457>.

Guo, Y., Lin, D., Zhang, M., Zhang, X., Li, Y., Yang, R., et al. (2016). CLDN6-induced apoptosis via regulating ASK1-p38/JNK signaling in breast cancer MCF-7 cells. *International Journal of Oncology* 48(6)**,** 2435-2444. doi: <https://doi.org/10.3892/ijo.2016.3469>.

Guo, Y., Wang, W., Wang, J., Feng, J., Wang, Q., Jin, J., et al. (2013). Receptor for Activated C Kinase 1 Promotes Hepatocellular Carcinoma Growth by Enhancing Mitogen-Activated Protein Kinase Kinase 7 Activity. *Hepatology* 57(1)**,** 140-151. doi: <https://doi.org/10.1002/hep.25978>.

Guo, Y., Xu, X., Liu, Z., Zhang, T., Zhang, X., Wang, L., et al. (2012). Apoptosis signal-regulating kinase 1 is associated with the effect of claudin-6 in breast cancer. *Diagnostic Pathology* 7**,** #111. doi: <https://doi.org/10.1186/1746-1596-7-111>.

Handra-Luca, A., Lesty, C., Hammel, P., Sauvanet, A., Rebours, V., Martin, A., et al. (2012). Biological and prognostic relevance of mitogen-activated protein kinases in pancreatic adenocarcinoma. *Pancreas* 41(3)**,** 416-421. doi: <https://doi.org/10.1097/MPA.0b013e318238379d>.

He, J., Jin, Y., Zhou, M., Li, X., Chen, W., Wang, Y., et al. (2018). Solute carrier family 35 member F2 is indispensable for papillary thyroid carcinoma progression through activation of transforming growth factor-β type I receptor/apoptosis signal-regulating kinase 1/mitogen-activated protein kinase signaling axis. *Cancer Science* 109**,** 642-655. doi: <https://doi.org/10.1111/cas.13478>.

Huang, F.-T., Peng, J.-F., Cheng, W.-J., Zhuang, Y.-Y., Wang, L.-Y., Li, C.-Q., et al. (2017a). MiR-143 Targeting TAK1 Attenuates Pancreatic Ductal Adenocarcinoma Progression via MAPK and NF-κB Pathway In Vitro. *Digestive Diseases and Sciences* 62**,** 944-957. doi: <https://doi.org/10.1007/s10620-017-4472-7>.

Huang, H.-L., Chiang, C.-H., Hung, W.-C., and Hou, M.-F. (2015). Targeting of TGF-β-activated protein kinase 1 inhibits chemokine (C-C motif) receptor 7 expression, tumor growth and metastasis in breast cancer. *Oncotarget* 6(2)**,** 995-1007. doi: <https://doi.org/10.18632/oncotarget.2739>.

Huang, H.L., Jin, H., Zhao, H., Wang, J., Li, X., Yan, H., et al. (2017b). RhoGDIβ promotes Sp1/MMP-2 expression and bladder cancer invasion through perturbing miR-200c-targeted JNK2 protein translation. *Molecular Oncology* 11**,** 1579-1594. doi: <https://doi.org/10.1002/1878-0261.12132>.

Iansante, V., Choy, P.M., Fung, S.W., Liu, Y., Chai, J.-G., Dyson, J., et al. (2015). PARP14 promotes the Warburg effect in hepatocellular carcinoma by inhibiting JNK1-dependent PKM2 phosphorylation and activation. *Nature Communications* 6**,** #7882. doi: <https://doi.org/10.1038/ncomms8882>.

Ida, L., Yamaguchi, T., Yanagisawa, K., Kajino, T., Shimada, Y., Suzuki, M., et al. (2016). Receptor tyrosine kinase-like orphan receptor 1, a target of *NKX2-1*/*TTF-1* lineage-survival oncogene, inhibits apoptosis signal-regulating kinase 1-mediated pro-apoptotic signaling in lung adenocarcinoma. *Cancer Science* 107(2)**,** 155-161. doi: <https://doi.org/10.1111/cas.12858>.

Iriondo, O., Liu, Y., Lee, G., Elhodaky, M., Jimenez, C., Li, L., et al. (2018). TAK1 mediates microenvironment-triggered autocrine signals and promotes triple-negative breast cancer lung metastasis. *Nature Communications* 9**,** #1994. doi: <https://doi.org/10.1038/s41467-018-04460-w>.

Ishaq, M., Kumar, S., Varinli, H., Han, Z.J., Rider, A.E., Evans, M.D.M., et al. (2014). Atmospheric gas plasma–induced ROS production activates TNF-ASK1 pathway for the induction of melanoma cancer cell apoptosis. *Molecular Biology of the Cell* 25(9)**,** 1523-1531. doi: <https://doi.org/10.1091/mbc.e13-10-0590>.

Jia, M., Zhu, M., Zhou, F., Wang, M., Sun, M., Yang, Y., et al. (2017). Genetic variants of JNK and p38α pathways and risk of non-small cell lung cancer in an Eastern Chinese population. *International Journal of Cancer* 140**,** 807-817. doi: <https://doi.org/10.1002/ijc.30508>.

Jiang, C.-F., Wen, L.-Z., Yin, C., Xu, W.-P., Shi, B., Zhang, X., et al. (2016). Apoptosis signal-regulating kinase 1 mediates the inhibitory effect of hepatocyte nuclear factor-4α on hepatocellular carcinoma. *Oncotarget* 7(19)**,** 27408-27421. doi: <https://doi.org/10.18632/oncotarget.8478>.

Kamiyama, M., Shirai, T., Tamura, S., Suzuki-Inoue, K., Ehata, S., Takahashi, K., et al. (2017). ASK1 facilitates tumor metastasis through phosphorylation of an ADP receptor P2Y_12_ in platelets. *Cell Death and Differentiation* 24**,** 2066-2076. doi: <https://doi.org/10.1038/cdd.2017.114>.

Kim, K.-Y., Kim, B.-C., Xu, Z., and Kim, S.-J. (2004). Mixed Lineage Kinase 3 (MLK3)-activated p38 MAP Kinase Mediates Transforming Growth Factor-β-induced Apoptosis in Hepatoma Cells. *The Journal of Biological Chemistry* 279(28)**,** 29478-29484. doi: <https://doi.org/10.1074/jbc.M313947200>.

Kitanaka, C., Sato, A., and Okada, M. (2013). JNK Signaling in the Control of the Tumor-Initiating Capacity Associated with Cancer Stem Cells. *Genes & Cancer* 4**,** 388-396. doi: <https://doi.org/10.1177/1947601912474892>.

Kluth, M., Hesse, J., Heinl, A., Krohn, A., Steurer, S., Sirma, H., et al. (2013). Genomic deletion of MAP3K7 at 6q12-22 is associated with early PSA recurrence in prostate cancer and absence of *TMPRSS2:ERG* fusions. *Modern Pathology* 26**,** 975-983. doi: <https://doi.org/10.1038/modpathol.2012.236>.

Kyriakis, J.M., and Avruch, J. (2012). Mammalian MAPK signal transduction pathways activated by stress and inflammation: a 10-year update. *Physiological Reviews* 92(2)**,** 689-737. doi: <https://doi.org/10.1152/physrev.00028.2011>.

Lam, C.R.I., Tan, C., Teo, Z., Tay, C.Y., Phua, T., Wu, Y.L., et al. (2013). Loss of TAK1 increases cell traction force in a ROS-dependent manner to drive epithelial–mesenchymal transition of cancer cells. *Cell Death & Disease* 4**,** e848. doi: <https://doi.org/10.1038/cddis.2013.339>.

Lessel, W., Silver, A., Jechorek, D., Guenther, T., Roehl, F.-W., Kalinski, T., et al. (2017). Inactivation of JNK2 as carcinogenic factor in colitis-associated and sporadic colorectal carcinogenesis. *Carcinogenesis* 38(5)**,** 559-569. doi: <https://doi.org/10.1093/carcin/bgx032>.

Li, L., and Luo, Z. (2017). Dysregulated miR-27a-3p promotes nasopharyngeal carcinoma cell proliferation and migration by targeting Mapk10. *Oncology Reports* 37(5)**,** 2679-2687. doi: <https://doi.org/10.3892/or.2017.5544>.

Li, Y.-S., Deng, Z.-H., Zeng, C., and Lei, G.-H. (2016). JNK pathway in osteosarcoma: pathogenesis and therapeutics. *Journal of Receptors and Signal Transduction* 36(5)**,** 465-470. doi: <https://doi.org/10.3109/10799893.2015.1122045>.

Lin, P., Niu, W., Peng, C., Zhang, Z., and Niu, J. (2015). The role of TAK1 expression in thyroid cancer. *International Journal of Clinical and Experimental Pathology* 8(11)**,** 14449-11456.

Lisanti, M.P., Reeves, K., Peiris-Pagès, M., Chadwick, A.L., Sanchez-Alvarez, R., Howell, A., et al. (2014). JNK1 stress signaling is hyper-activated in high breast density and the tumor stroma: Connecting fibrosis, inflammation, and stemness for cancer prevention. *Cell Cycle* 13(4)**,** 580-599. doi: <https://doi.org/10.4161/cc.27379>.

Liu, J., Wang, T., Creighton, C.J., Wu, S.-P., Ray, M., Janardhan, K.S., et al. (2019). JNK^1/2^ represses *Lkb^1^*-deficiency-induced lung squamous cell carcinoma progression. *Nature Communications* 10(1)**,** #2148. doi: <https://doi.org/10.1038/s41467-019-09843-1>.

Liu, Y., Zhang, X., Wang, J., Yang, J., and Tan, W. (2015). JNK is required for maintaining the tumor-initiating cell-like properties of acquired chemoresistant human cancer cells. *Acta Pharmacologica Sinica* 36**,** 1099-1106. doi: <https://doi.org/10.1038/aps.2015.58>.

Lu, J., Zhou, L., Yang, G., Liang, Z.-Y., Zhou, W.-X., You, L., et al. (2019). Clinicopathological and prognostic significance of MKK4 and MKK7 in resectable pancreatic ductal adenocarcinoma. *Human Pathology* 86**,** 143-154. doi: <https://doi.org/10.1016/j.humpath.2018.11.026>.

Lu, K.-H., Su, S.-C., Lin, C.-W., Hsieh, Y.-H., Lin, Y.-C., Chien, M.-H., et al. (2018). Melatonin attenuates osteosarcoma cell invasion by suppression of C-C motif chemokine ligand 24 through inhibition of the c-Jun N-terminal kinase pathway. *Journal of Pineal Research* 65**,** e12507. doi: <https://doi.org/10.1111/jpi.12507>.

Martini, M., Russo, M., Lamba, S., Vitiello, E., Crowley, E.H., Sassi, F., et al. (2013). Mixed lineage kinase MLK4 is activated in colorectal cancers where it synergistically cooperates with activated RAS signaling in driving tumorigenesis. *Cancer Research* 73(6)**,** 1912-1921. doi: <https://doi.org/10.1158/0008-5472.Can-12-3074>.

Marusiak, A.A., Prelowska, M.K., Mehlich, D., Lazniewski, M., Kaminska, K., Gorczynski, A., et al. (2019). Upregulation of MLK4 promotes migratory and invasive potential of breast cancer cells. *Oncogene* 38**,** 2860-2875. doi: <https://doi.org/10.1038/s41388-018-0618-0>.

Marusiak, A.A., Stephenson, N.L., Baik, H., Trotter, E.W., Li, Y., Blyth, K., et al. (2016). Recurrent MLK4 Loss-of-Function Mutations Suppress JNK Signaling to Promote Colon Tumorigenesis. *Cancer Research* 76(3)**,** 724-735. doi: <https://doi.org/10.1158/0008-5472.CAN-15-0701-T>.

Melisi, D., Xia, Q., Paradiso, G., Ling, J., Moccia, T., Carbone, C., et al. (2011). Modulation of pancreatic cancer chemoresistance by inhibition of TAK1. *Journal of the National Cancer Institute* 103(15)**,** 1190-1204. doi: <https://doi.org/10.1093/jnci/djr243>.

Min, J., Huang, K., Tang, H., Ding, X., Qi, C., Qin, X., et al. (2015). Phloretin induces apoptosis of non-small cell lung carcinoma A549 cells via JNK1/2 and p38 MAPK pathways. *Oncology Reports* 34**,** 2871-2879. doi: <https://doi.org/10.3892/or.2015.4325>.

Misek, S.A., Chen, J., Schroeder, L., Rattanasinchai, C., Sample, A., Sarkaria, J.N., et al. (2017). EGFR Signals through a DOCK180-MLK3 Axis to Drive Glioblastoma Cell Invasion. *Molecular Cancer Research* 15(8)**,** 1085-1095. doi: <https://doi.org/10.1158/1541-7786.MCR-16-0318>.

Mundt, F., Rajput, S., Li, S., Ruggles, K.V., Mooradian, A.D., Mertins, P., et al. (2018). Mass Spectrometry-Based Proteomics Reveals Potential Roles of NEK9 and MAP2K4 in Resistance to PI3K Inhibition in Triple-Negative Breast Cancers. *Cancer Research* 78(10)**,** 2732-2746. doi: <https://doi.org/10.1158/0008-5472.Can-17-1990>.

Neil, J.R., and Schiemann, W.P. (2008). Altered TAB1:I κB kinase interaction promotes transforming growth factor β-mediated nuclear factor-κB activation during breast cancer progression. *Cancer Research* 68(5)**,** 1462-1470. doi: <https://doi.org/10.1158/0008-5472.Can-07-3094>.

Nie, F., Liu, T., Zhong, L., Yang, X., Liu, Y., Xia, H., et al. (2016). MicroRNA-148b enhances proliferation and apoptosis in human renal cancer cells via directly targeting MAP3K9. *Molecular Medicine Reports* 13**,** 83-90. doi: <https://doi.org/10.3892/mmr.2015.4555>.

Nitta, R.T., Del Vecchio, C.A., Chu, A.H., Mitra, S.S., Godwin, A.K., and Wong, A.J. (2011). The role of the c-Jun N-terminal kinase 2-α-isoform in non-small cell lung carcinoma tumorigenesis. *Oncogene* 30**,** 234-244. doi: <https://doi.org/10.1038/onc.2010.414>.

Okada, M., Shibuya, K., Sato, A., Seino, S., Suzuki, S., Seino, M., et al. (2014). Targeting the K-Ras - JNK axis eliminates cancer stem-like cells and prevents pancreatic tumor formation. *Oncotarget* 5(13)**,** 5100-5112. doi: <https://doi.org/10.18632/oncotarget.2087>.

Okada, M., Shibuya, K., Sato, A., Seino, S., Watanabe, E., Suzuki, S., et al. (2013). Specific role of JNK in the maintenance of the tumor-initiating capacity of A549 human non-small cell lung cancer cells. *Oncology Reports* 30(4)**,** 1957-1964. doi: <https://doi.org/10.3892/or.2013.2655>.

Pan, W., Wang, H., Jianwei, R., and Ye, Z. (2014). MicroRNA-27a promotes proliferation, migration and invasion by targeting *MAP2K4* in human osteosarcoma cells. *Cellular Physiology and Biochemistry* 33**,** 402-412. doi: <https://doi.org/10.1159/000356679>.

Park, J.G., Aziz, N., and Cho, J.Y. (2019). MKK7, the essential regulator of JNK signaling involved in cancer cell survival: a newly emerging anticancer therapeutic target. *Therapeutic Advances in Medical Oncology* 11**,** 1-14. doi: <https://doi.org/10.1177/1758835919875574>.

Parra, E., and Ferreira, J. (2013). Modulation of the response of prostate cancer cell lines to cisplatin treatment using small interfering RNA. *Oncology Reports* 30(4)**,** 1936-1942. doi: <https://doi.org/10.3892/or.2013.2637>.

Pavese, J.M., Ogden, I.M., Voll, E.A., Huang, X., Xu, L., Jovanovic, B., et al. (2014). Mitogen-Activated Protein Kinase Kinase 4 (MAP2K4) Promotes Human Prostate Cancer Metastasis. *PLoS ONE* 9(7)**,** e102289. doi: <https://doi.org/10.1371/journal.pone.0102289>.

Prickett, T.D., Zerlanko, B., Gartner, J.J., Parker, S.C.J., Dutton-Regester, K., Lin, J.C., et al. (2014). Somatic Mutations in MAP3K5 Attenuate Its Proapoptotic Function in Melanoma through Increased Binding to Thioredoxin. *Journal of Investigative Dermatology* 134(2)**,** 452-460. doi: <https://doi.org/10.1038/jid.2013.365>.

Qiu, F., Yang, L., Lu, X., Chen, J., Wu, D., Wei, Y., et al. (2016). The *MKK7* p.Glu116Lys Rare Variant Serves as a Predictor for Lung Cancer Risk and Prognosis in Chinese. *PLoS Genetics* 12(3)**,** e1005955. doi: <https://doi.org/10.1371/journal.pgen.1005955>.

Reissig, K., Silver, A., Hartig, R., Schinlauer, A., Walluscheck, D., Guenther, T., et al. (2017). Chk1 Promotes DNA Damage Response Bypass following Oxidative Stress in a Model of Hydrogen Peroxide-Associated Ulcerative Colitis through JNK Inactivation and Chromatin Binding. *Oxidative Medicine and Cellular Longevity* 2017**,** #9303158. doi: <https://doi.org/10.1155/2017/9303158>.

Rodrigues, L.U., Rider, L., Nieto, C., Romero, L., Karimpour-Fard, A., Loda, M., et al. (2015). Coordinate loss of MAP3K7 and CHD1 promotes aggressive prostate cancer. *Cancer Research* 75(6)**,** 1021-1034. doi: <https://doi.org/10.1158/0008-5472.Can-14-1596>.

Safina, A., Ren, M.Q., Vandette, E., and Bakin, A.V. (2008). TAK1 is required for TGF-β1-mediated regulation of matrix metalloproteinase-9 and metastasis. *Oncogene* 27**,** 1198-1207. doi: <https://doi.org/10.1038/sj.onc.1210768>.

Safina, A., Sotomayor, P., Limoge, M., Morrison, C., and Bakin, A.V. (2011). TAK1-TAB2 signaling contributes to bone destruction by breast carcinoma cells. *Molecular Cancer Research* 9(8)**,** 1042-1053. doi: <https://doi.org/10.1158/1541-7786.Mcr-10-0196>.

Sakai, H., Sato, A., Aihara, Y., Ikarashi, Y., Midorikawa, Y., Kracht, M., et al. (2014). MKK7 mediates miR-493-dependent suppression of liver metastasis of colon cancer cells. *Cancer Science* 105(4)**,** 425-430. doi: <https://doi.org/10.1111/cas.12380>.

Schroyer, A.L., Stimes, N.W., Abi Saab, W.F., and Chadee, D.N. (2018). MLK3 phosphorylation by ERK1/2 is required for oxidative stress-induced invasion of colorectal cancer cells. *Oncogene* 37**,** 1031-1040. doi: <https://doi.org/10.1038/onc.2017.396>.

Singh, A., Sweeney, M.F., Yu, M., Burger, A., Greninger, P., Benes, C., et al. (2012). TAK1 Inhibition Promotes Apoptosis in KRAS-Dependent Colon Cancers. *Cell* 148(4)**,** 639-650. doi: <https://doi.org/10.1016/j.cell.2011.12.033>.

Sobhan, P.K., Zhai, Q., Green, L.C., Hansford, L.M., and Funa, K. (2017). ASK1 regulates the survival of neuroblastoma cells by interacting with TLX and stabilizing HIF-1α. *Cellular Signalling* 30**,** 104-117. doi: <https://doi.org/10.1016/j.cellsig.2016.11.018>.

Stark, M.S., Woods, S.L., Gartside, M.G., Bonazzi, V.F., Dutton-Regester, K., Aoude, L.G., et al. (2011). Frequent somatic mutations in *MAP3K5* and *MAP3K9* in metastatic melanoma identified by exome sequencing. *Nature Genetics* 44(2)**,** 165-169. doi: <https://doi.org/10.1038/ng.1041>.

Stephens, P.J., Tarpey, P.S., Davies, H., Van Loo, P., Greenman, C., Wedge, D.C., et al. (2012). The landscape of cancer genes and mutational processes in breast cancer. *Nature* 486**,** 400-404. doi: <https://doi.org/10.1038/nature11017>.

Sui, H., Cai, G.X., Pan, S.F., Deng, W.L., Wang, Y.W., Chen, Z.S., et al. (2014). miR200c Attenuates P-gp-Mediated MDR and Metastasis by Targeting JNK2/c-Jun Signaling Pathway in Colorectal Cancer. *Molecular Cancer Therapeutics* 13(12)**,** 3137-3151. doi: <https://doi.org/10.1158/1535-7163.Mct-14-0167>.

Sun, M., Isaacs, G.D., Hah, N., Heldring, N., Fogarty, E.A., and Kraus, W.L. (2012). Estrogen Regulates JNK1 Genomic Localization to Control Gene Expression and Cell Growth in Breast Cancer Cells. *Molecular Endocrinology* 26(5)**,** 736-747. doi: <https://doi.org/10.1210/me.2011-1158>.

Tang, B., Du, J., Wang, J., Tan, G., Gao, Z., Wang, Z., et al. (2012). Alpinetin suppresses proliferation of human hepatoma cells by the activation of MKK7 and elevates sensitization to cis-diammined dichloridoplatium. *Oncology Reports* 27(4)**,** 1090-1096. doi: <https://doi.org/10.3892/or.2011.1580>.

Tesser-Gamba, F., Petrilli, A.S., de Seixas Alves, M.T., Filho, R.J.G., Juliano, Y., and Toledo, S.R.C. (2012). *MAPK7* and *MAP2K4* as prognostic markers in osteosarcoma. *Human Pathology* 43(7)**,** 994-1002. doi: <https://doi.org/10.1016/j.humpath.2011.08.003>.

Tey, S.K., Tse, E.Y.T., Mao, X., Ko, F.C.F., Wong, A.S.T., Lo, R.C.-L., et al. (2017). Nuclear Met promotes hepatocellular carcinoma tumorigenesis and metastasis by upregulation of TAK1 and activation of NF-κB pathway. *Cancer Letters* 411**,** 150-161. doi: <https://doi.org/10.1016/j.canlet.2017.09.047>.

Tornatore, L., Sandomenico, A., Raimondo, D., Low, C., Rocci, A., Tralau-Stewart, C., et al. (2014). Cancer-selective targeting of the NF-κB survival pathway with GADD45β/MKK7 inhibitors. *Cancer Cell* 26(4)**,** 495-508. doi: <https://doi.org/10.1016/j.ccr.2014.07.027>.

Tournier, C. (2013). The 2 Faces of JNK Signaling in Cancer. *Genes & Cancer* 4**,** 397-400. doi: <https://doi.org/10.1177/1947601913486349>.

Velho, S., Oliveira, C., Paredes, J., Sousa, S., Leite, M., Matos, P., et al. (2010). Mixed lineage kinase 3 gene mutations in mismatch repair deficient gastrointestinal tumours. *Human Molecular Genetics* 19(4)**,** 697-706. doi: <https://doi.org/10.1093/hmg/ddp536>.

Velho, S., Pinto, A., Licastro, D., Oliveira, M.J., Sousa, F., Stupka, E., et al. (2014). Dissecting the signaling pathways associated with the oncogenic activity of MLK3 P252H mutation. *BMC Cancer* 14**,** #182. doi: <https://doi.org/10.1186/1471-2407-14-182>.

Wagner, E.F., and Nebreda, Á.R. (2009). Signal integration by JNK and p38 MAPK pathways in cancer development. *Nature Reviews Cancer* 9(8)**,** 537-549. doi: <https://doi.org/10.1038/nrc2694>.

Wan, X., Huang, W., Yang, S., Zhang, Y., Zhang, P., Kong, Z., et al. (2016). Androgen-induced miR-27A acted as a tumor suppressor by targeting MAP2K4 and mediated prostate cancer progression. *The International Journal of Biochemistry & Cell Biology* 79**,** 249-260. doi: <https://doi.org/10.1016/j.biocel.2016.08.043>.

Wan, X., Shi, L., Ma, X., Tang, H., and Wu, G. (2018). The Elevated ASK1 Expression Inhibits Proliferation and Invasion in Gastric Cancer HGC-27 Cells. *The Anatomical Record* 301(11)**,** 1815-1819. doi: <https://doi.org/10.1002/ar.23906>.

Wang, C., Zhang, X., Zhang, C., Zhai, F., Li, Y., and Huang, Z. (2017a). MicroRNA-155 targets MAP3K10 and regulates osteosarcoma cell growth. *Pathology - Research and Practice* 213(4)**,** 389-393. doi: <https://doi.org/10.1016/j.prp.2016.12.028>.

Wang, H., Sun, X., Gao, F., Zhong, B., Zhang, Y., and Sun, Z. (2012). Effect of ulinastatin on growth inhibition, apoptosis of breast carcinoma cells is related to a decrease in signal conduction of JNk-2 and NF-κB. *Journal of Experimental & Clinical Cancer Research* 31**,** #2. doi: <https://doi.org/10.1186/1756-9966-31-2>.

Wang, J., and Tai, G. (2016). Role of C-Jun N-terminal Kinase in Hepatocellular Carcinoma Development. *Targeted Oncology* 11**,** 723-738. doi: <https://doi.org/10.1007/s11523-016-0446-5>.

Wang, P.-N., Huang, J., Duan, Y.-H., Zhou, J.-M., Huang, P.-Z., Fan, X.-J., et al. (2017b). Downregulation of phosphorylated MKK4 is associated with a poor prognosis in colorectal cancer patients. *Oncotarget* 8(21)**,** 34352-34361. doi: <https://doi.org/10.18632/oncotarget.16128>.

Wang, Y.-D., Chen, W.-D., Li, C., Guo, C., Li, Y., Qi, H., et al. (2015). Farnesoid X receptor antagonizes JNK signaling pathway in liver carcinogenesis by activating *SOD3*. *EMolecular Endocrinology* 29(2)**,** 322-331. doi: <https://doi.org/10.1210/me.2014-1225>.

Wei, C., Lai, Y.-Q., Li, X.-X., and Ye, J.-X. (2013). TGF-β-activated kinase-1: a potential prognostic marker for clear cell renal cell carcinoma. *Asian Pacific Journal of Cancer Prevention* 14(1)**,** 315-320. doi: <https://doi.org/10.7314/apjcp.2013.14.1.315>.

Wen, J., Hu, Y., Luo, K.-J., Yang, H., Zhang, S.-S., and Fu, J.-H. (2013). Positive Transforming Growth Factor-β Activated Kinase-1 Expression Has an Unfavorable Impact on Survival in T3N1-3M0 Esophageal Squamous Cell Carcinomas. *The Annals of Thoracic Surgery* 95(1)**,** 285-290. doi: <https://doi.org/10.1016/j.athoracsur.2012.09.050>.

Whitworth, H., Bhadel, S., Ivey, M., Conaway, M., Spencer, A., Hernan, R., et al. (2012). Identification of kinases regulating prostate cancer cell growth using an RNAi phenotypic screen. *PLoS ONE* 7(6)**,** e38950. doi: <https://doi.org/10.1371/journal.pone.0038950>.

Wu, M., Shi, L., Cimic, A., Romero, L., Sui, G., Lees, C.J., et al. (2012). Suppression of Tak1 promotes prostate tumorigenesis. *Cancer Research* 72(11)**,** 2833-2843. doi: <https://doi.org/10.1158/0008-5472.Can-11-2724>.

Wu, Q., Wu, W., Fu, B., Shi, L., Wang, X., and Kuca, K. (2019). JNK signaling in cancer cell survival. *Medical Research Reviews* 39**,** 2082-2104. doi: <https://doi.org/10.1002/med.21574>.

Wu, X., Gong, Z., Sun, L., Ma, L., and Wang, Q. (2017). MicroRNA-802 plays a tumour suppressive role in tongue squamous cell carcinoma through directly targeting MAP2K4. *Cell Proliferation* 50**,** e12336. doi: <https://doi.org/10.1111/cpr.12336>.

Wu, X., Zhang, W., Font-Burgada, J., Palmer, T., Hamil, A.S., Biswas, S.K., et al. (2014). Ubiquitin-conjugating enzyme Ubc13 controls breast cancer metastasis through a TAK1-p38 MAP kinase cascade. *Proceedings of the National Academy of Sciences of the United States of America* 111(38)**,** 13870-13875. doi: <https://doi.org/10.1073/pnas.1414358111>.

Wu, Y.-H., Ai, X., Liu, F.-Y., Liang, H.-F., Zhang, B.-X., and Chen, X.-P. (2016). c-Jun N-terminal kinase inhibitor favors transforming growth factor-β to antagonize hepatitis B virus X protein-induced cell growth promotion in hepatocellular carcinoma. *Molecular Medicine Reports* 13(2)**,** 1345-1352. doi: <https://doi.org/10.3892/mmr.2015.4644>.

Xie, X., Kaoud, T.S., Edupuganti, R., Zhang, T., Kogawa, T., Zhao, Y., et al. (2017). c-Jun N-terminal kinase promotes stem cell phenotype in triple-negative breast cancer through upregulation of Notch1 via activation of c-Jun. *Oncogene* 36**,** 2599-2608. doi: <https://doi.org/10.1038/onc.2016.417>.

Xu, R., and Hu, J. (2020). The role of JNK in prostate cancer progression and therapeutic strategies. *Biomedicine & Pharmacotherapy* 121**,** #109679. doi: <https://doi.org/10.1016/j.biopha.2019.109679>.

Yan, D., An, G., and Kuo, M.T. (2016). C-Jun N-terminal kinase signalling pathway in response to cisplatin. *Journal of Cellular and Molecular Medicine* 20**,** 2013-2019. doi: <https://doi.org/10.1111/jcmm.12908>.

Yang, Y., Qiu, Y., Tang, M., Wu, Z., Hu, W., and Chen, C. (2017). Expression and function of transforming growth factor-β-activated protein kinase 1 in gastric cancer. *Molecular Medicine Reports* 16(3)**,** 3103-3110. doi: <https://doi.org/10.3892/mmr.2017.6998>.

Yeasmin, S., Nakayama, K., Rahman, M.T., Rahman, M., Ishikawa, M., Katagiri, A., et al. (2011). MKK4 acts as a potential tumor suppressor in ovarian cancer. *Tumor Biology* 32(4)**,** 661-670. doi: <https://doi.org/10.1007/s13277-011-0166-5>.

Yoo, K.H., Park, Y.-K., Kim, H.-S., Jung, W.-W., and Chang, S.-G. (2011). Identification of MAPK10 as a novel epigenetic marker for chromophobe kidney cancer. *Pathology International* 61**,** 52-54. doi: <https://doi.org/10.1111/j.1440-1827.2010.02605.x>.

Zhan, Y., Abi Saab, W.F., Modi, N., Stewart, A.M., Liu, J., and Chadee, D.N. (2012). Mixed lineage kinase 3 is required for matrix metalloproteinase expression and invasion in ovarian cancer cells. *Experimental Cell Research* 318(14)**,** 1641-1648. doi: <https://doi.org/10.1016/j.yexcr.2012.05.002>.

Zhang, L., He, L., Zhang, H., and Chen, Y. (2018). Knockdown of MiR-20a Enhances Sensitivity of Colorectal Cancer Cells to Cisplatin by Increasing ASK1 Expression. *Cellular Physiology and Biochemistry* 47(4)**,** 1432-1441. doi: <https://doi.org/10.1159/000490834>.

Zhang, N., Liu, L., Dou, Y., Song, D., and Deng, H. (2016). Glycogen synthase kinase-3β antagonizes ROS-induced hepatocellular carcinoma cell death through suppression of the apoptosis signal-regulating kinase 1. *Medical Oncology* 33**,** #60. doi: <https://doi.org/10.1007/s12032-016-0776-2>.

Zhang, S., Shi, L., Ma, H., Li, H., Li, Y., Lu, Y., et al. (2017). Dihydroartemisinin induces apoptosis in human gastric cancer cell line BGC-823 through activation of JNK1/2 and p38 MAPK signaling pathways. *Journal of Receptors and Signal Transduction* 37(2)**,** 174-180. doi: <https://doi.org/10.1080/10799893.2016.1203942>.

Zhang, X., Ruan, Y., Li, Y., Lin, D., Liu, Z., and Quan, C. (2015). Expression of apoptosis signal-regulating kinase 1 is associated with tight junction protein claudin-6 in cervical carcinoma. *International Journal of Clinical and Experimental Pathology* 8(5)**,** 5535-5541.

Zheng, R., You, Z., Jia, J., Lin, S., Han, S., Liu, A., et al. (2016). Curcumin enhances the antitumor effect of ABT-737 via activation of the ROS-ASK1-JNK pathway in hepatocellular carcinoma cells. *Molecular Medicine Reports* 13(2)**,** 1570-1576. doi: <https://doi.org/10.3892/mmr.2015.4715>.

Zhu, M.M., Tong, J.L., Xu, Q., Nie, F., Xu, X.T., Xiao, S.D., et al. (2012). Increased JNK1 Signaling Pathway Is Responsible for ABCG2-Mediated Multidrug Resistance in Human Colon Cancer. *PLoS ONE* 7(8)**,** e41763. doi: <https://doi.org/10.1371/journal.pone.0041763>.

Zhu, T., Wang, J., Pei, Y., Wang, Q., Wu, Y., Qiu, G., et al. (2016). Neddylation controls basal MKK7 kinase activity in breast cancer cells. *Oncogene* 35**,** 2624-2633. doi: <https://doi.org/10.1038/onc.2015.323>.
